# Supplementary material for: The Initiator Methionine tRNA Drives Secretion of Type II Collagen from Stromal Fibroblasts to Promote Tumor Growth and Angiogenesis
Source: Curr Biol. 2016 Mar 21;26(6):755–65. doi: 10.1016/j.cub.2016.01.045 (PMC4819511; doi:10.1016/j.cub.2016.01.045)
Supplement: Document S1. Figures S1–S7 and Supplemental Experimental Procedures [file mmc1.pdf]

Current Biology, Volume *26*

Supplemental Information

The Initiator Methionine tRNA Drives Secretion of Type II Collagen from Stromal Fibroblasts to Promote Tumor Growth and Angiogenesis

Cassie J. Clarke, Tracy J. Berg, Joanna Birch, Darren Ennis, Louise Mitchell, Catherine Cloix, Andrew Campbell, David Sumpton, Colin Nixon, Kirsteen Campbell, Victoria L. Bridgeman, Peter B. Vermeulen, Shane Foo, Eleftherios Kostaras, J. Louise Jones, Linda Haywood, Ellie Pulleine, Huabing Yin, Douglas Strathdee, Owen Sansom, Karen Blyth, Iain McNeish, Sara Zanivan, Andrew R. Reynolds, and Jim C. Norman

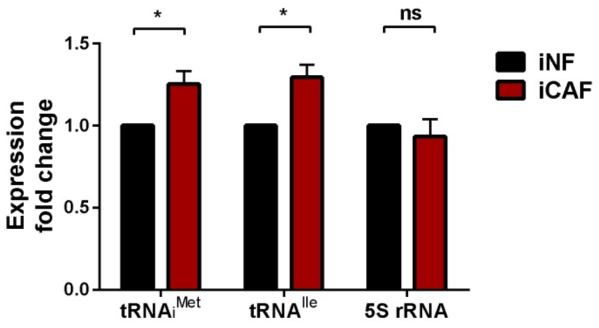

Figure S1. Changes in expression of specific tRNAs in cancer associated fibroblasts compared to normal fibroblasts.

qPCR was used to quantify expression of $tRNA_i^{Met}$, $tRNA^{Ile}$, and 5S rRNA in immortalised normal fibroblasts (iNF) and immortalised cancer associated fibroblasts (iCAF). All samples were normalised to ARPP P0 and are presented relative to iNF, n=minimum of 3, ± SEM, unpaired t-test, * $p < 0.05$.

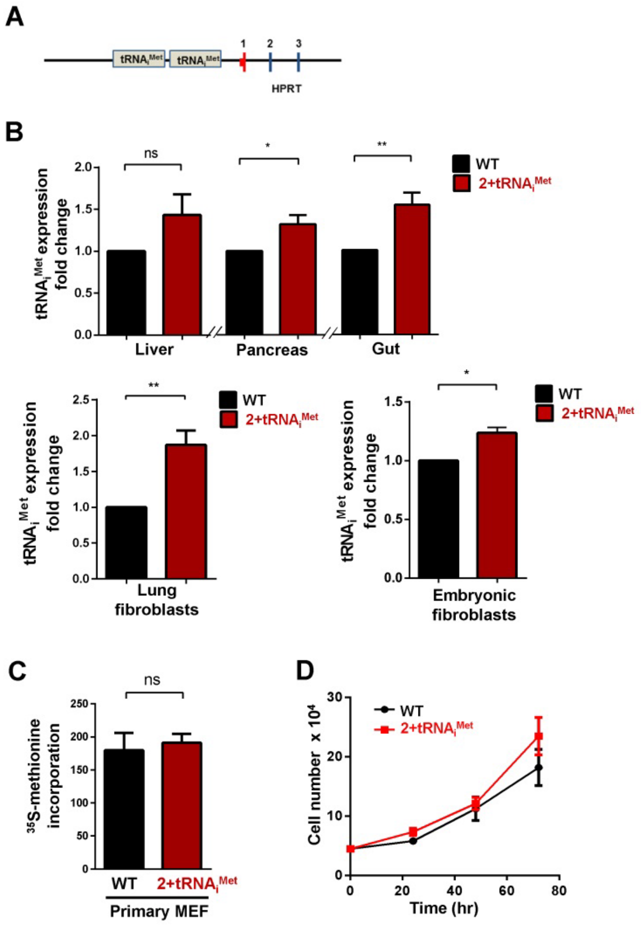

Figure S2. Characterisation of cells from $2{+}tRNA_i^{Met}$ mouse (related to Figure 2)

(A) The $2{+}tRNA_i^{Met}$ mouse was engineered to express two additional copies of the $tRNA_i^{Met}$ transgene in the HPRT locus on the X-chromosome.

(B) qPCR of $tRNA_i^{Met}$ expression in primary fibroblasts isolated from the lungs and embryos of the $2{+}tRNA_i^{Met}$ mouse. All values are normalised to expression of ARPP P0, n=4 lung and n=2 embryonic fibroblasts, ± SEM, unpaired t-test, * $p < 0.05$. ** $p < 0.005$.

(C) ^{35}S-methionine incorporation was used to assess the synthesis of new proteins. Primary cultured MEFs were depleted of methionine and cysteine for 30 minutes at 37°C/5% CO_2, then trypsinised and resuspended in media containing 0.07mCi EasyTag Express Protein Labelling Max ^{35}S and incubated at 37°C/5% CO_2 for 2 hours. TCA precipitation was used to stop and concentrate the reaction, and the ratio of TCA precipitated radiolabel to total radiolabel calculated, values are mean ± SEM, n=5.

(D) Primary cultured MEFs were isolated from wild-type and $2{+}tRNA_i^{Met}$ embryos, plated onto plastic dishes and their proliferation rate was determined over an 72 hr period. Values are mean ± SEM, n=2.

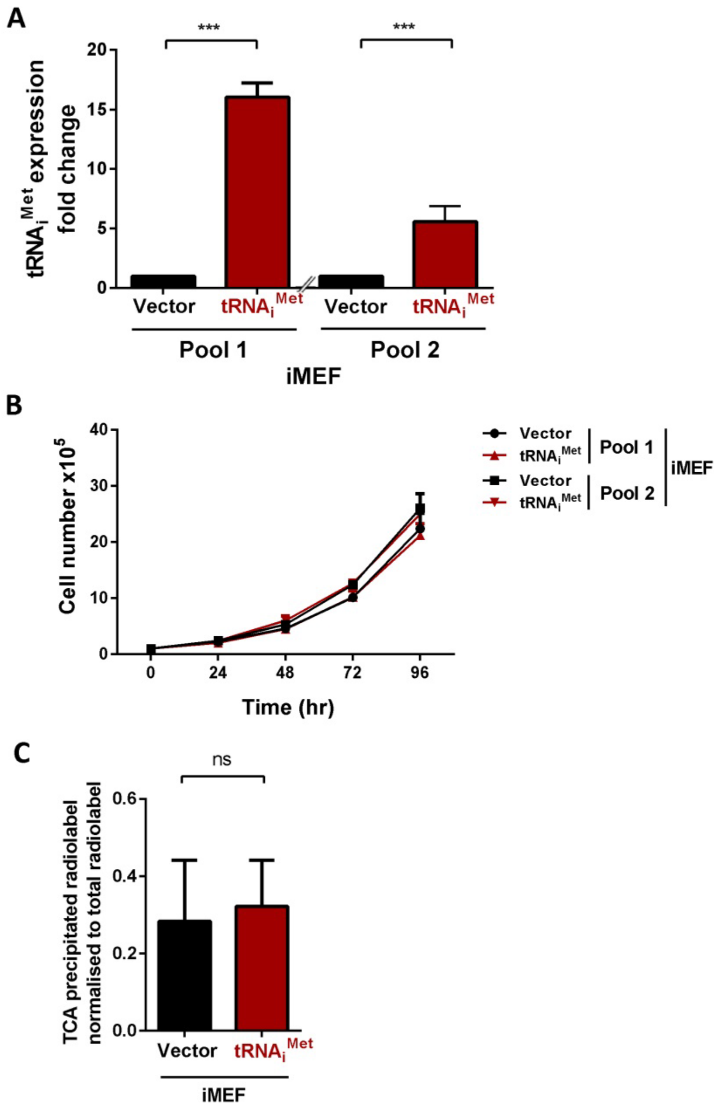

Figure S3. Characterisation of $tRNA_i^{Met}$ overexpressing fibroblasts (related to Figures 3 & 4)

(A) qPCR of $tRNA_i^{Met}$ expression in pools of immortalised mouse embryonic fibroblasts. Values are normalised to expression of ARPP P0, n=3, ± SEM, unpaired t-test, *** p <0.0005.

(B) Two pools of immortalised iMEFs which were overexpressing $tRNA_i^{Met}$ or vector control were plated onto plastic dishes and their proliferation rate was determined over an 96 hr period. Values are mean ± SEM, n=3.

(C) Immortalised MEFs which were overexpressing $tRNA_i^{Met}$ or vector control were labelled with ^{35}S-methionine and the incorporation of the tracer into newly synthesised protein was determined as for Figure S2C. Values are mean ± SEM, n=5.

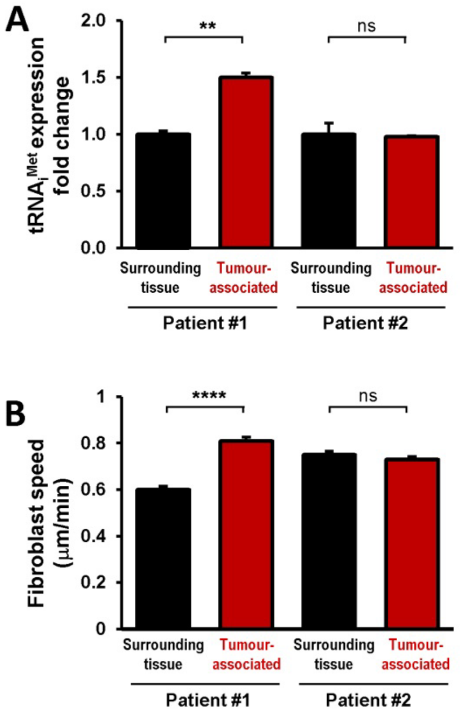

Figure S4. The relationship between tRNA$_i^{Met}$ levels and the migratory properties of ECM deposited by primary cultured CAFs.

Carcinoma-associated fibroblasts (CAFs) and control fibroblasts from surrounding normal tissue were isolated from resected material from two breast cancer patients (patient #1 and patient #2). qPCR was used to quantify tRNA$_i^{Met}$ levels (A). ECM was generated from these two pairs of carcinoma associated and normal fibroblasts and iMEFs were plated onto these. The speed of fibroblast migration on these ECMs was determined (B). Values are mean ± SEM, unpaired t-test, *** p <0.0001, ** p <0.001.

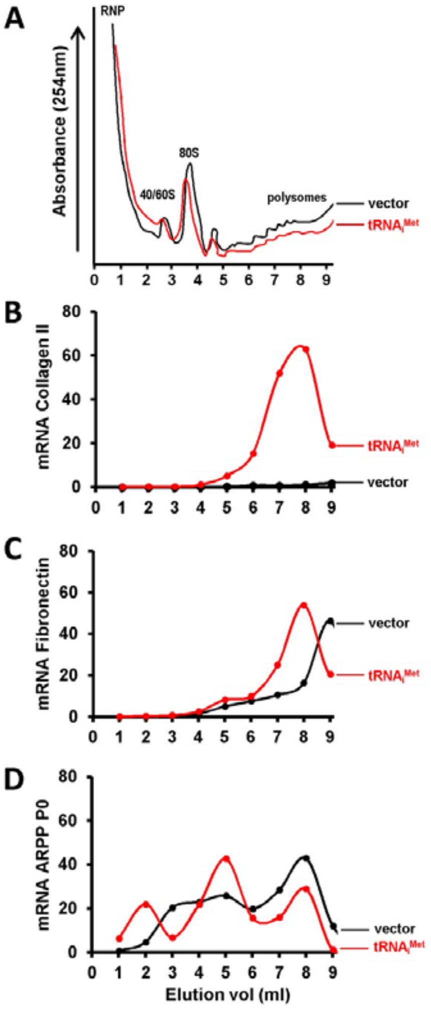

Figure S5. Polysome analysis of tRNA$_i^{Met}$ overexpressing fibroblasts (related to Figure 4)

iMEF-vector and iMEF-tRNA$_i^{Met}$ cells were treated with cycloheximide for 3 min and then lysed on ice. Lysates were centrifuged and loaded onto sucrose density gradients and centrifuged at 38,000rpm for 2 hr. Fractions were eluted (A) from the gradient and the presence of mRNAs for collagen II (B), fibronectin (C) and ARPP P0 (D) were determined using qPCR.

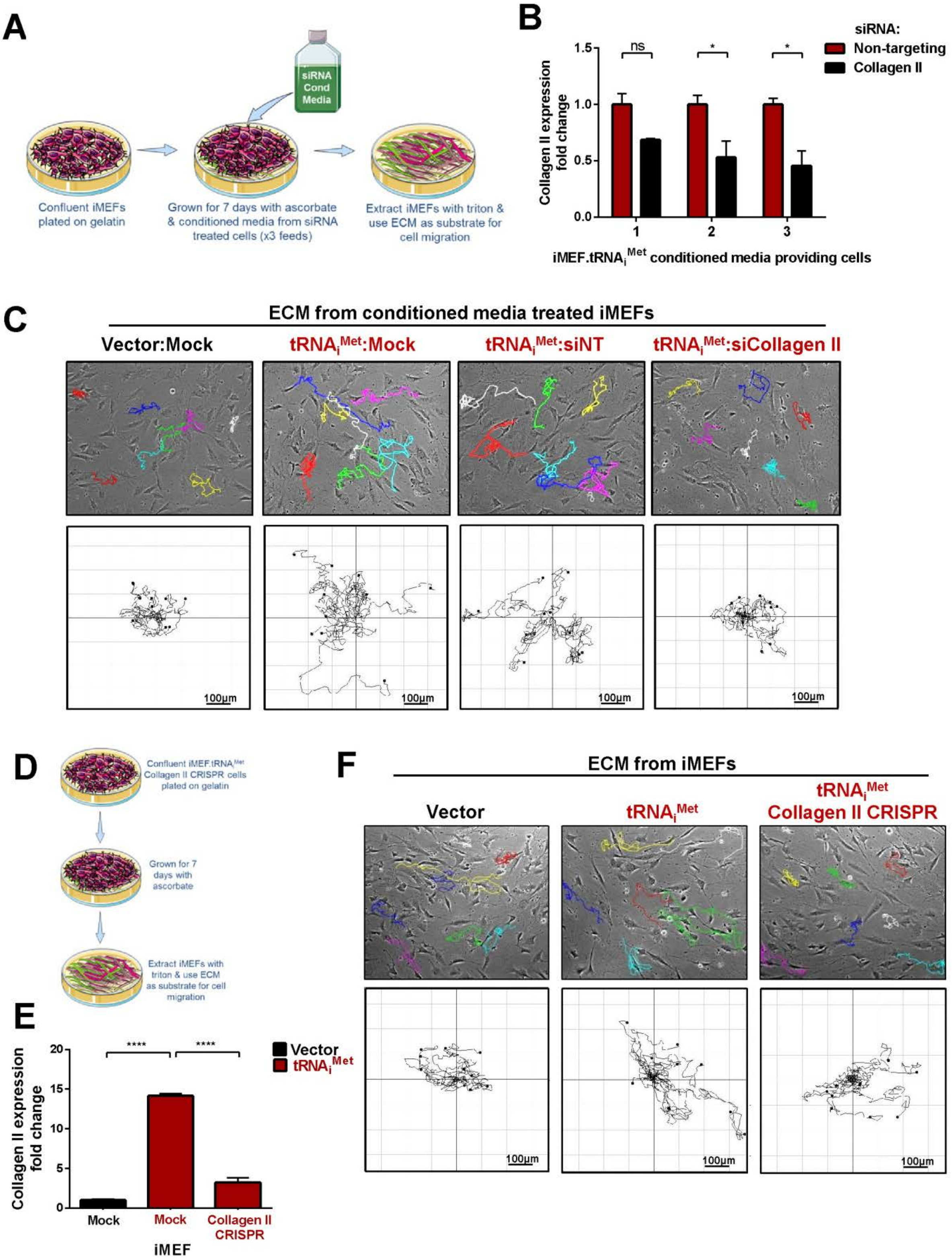

Figure S6. Type II Collagen secretion is required for tRNA$_i^{Met}$ to drive production of a pro-tumourigenic ECM (related to Figure 6)

(A) Schematic representation of the method used to generate conditioned media-derived ECM. iMEF cells were plated to confluence on gelatin-coated plates, and then grown for a further 7 days in ascorbate containing media that was diluted 1:1 with filter-sterilised conditioned media from iMEF.tRNA$_i^{Met}$ cells treated with either non-targeting or type II collagen-specific siRNA. The ascorbate media/conditioned media mix was refreshed and replaced every 2 days over a 7 day period. Cells were then removed using a triton containing buffer, leaving the ECM of interest coating the culture dish.

(B) qPCR was used to quantify type II collagen knockdown in the cells providing conditioned media to generate ECM. Values are normalised to expression of ARPP P0 and presented relative to expression in the iMEF.tRNA$_i^{Met}$ non-targeting siRNA-treated cells, values are mean ± SD, n=3, *p<0.05.

(C) Representative cell tracks and spider plots corresponding to the data displayed in Figure 5A.

(D) Schematic representation of method used to generate ECM from iMEF pools. iMEF cells were plated to confluence on gelatin-coated plates, and then grown for a further 7 days in ascorbate containing media. The ascorbate containing media was refreshed and replaced every 2 days over a 7 day period. Cells were then removed using a triton containing buffer, leaving the ECM of interest coating the culture dish.

(E) qPCR was used to quantify type II collagen levels in iMEF-vector and iMEF-tRNA$_i^{Met}$ cells transfected with an empty CRISPR vector, and in iMEF-tRNA$_i^{Met}$ cells transfected with a CRISPR vector specific for type II collagen. Values are normalised to expression of ARPP P0 and presented relative to expression in the iMEF.vector cells.

(F) Representative cell tracks and spider plots corresponding to the data displayed in Figure 5C.

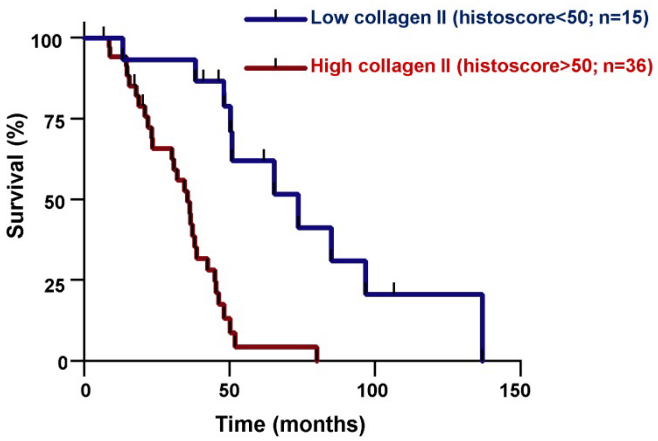

Figure S7. Type II collagen expression predicts poor prognosis in high grade serous ovarian cancer (related to Figure 7)

A tissue microarray (TMA) containing 51 cases of high grade serous ovarian cancer was stained for type II collagen and analysed as for Figure 7. The Kaplan-Meier analysis patient survival when the cohort is divided into tumours with low (histoscore < 50) and high (histoscore >50) collagen II expression. Patients with tumours displaying a collagen II histoscore greater than 50 have a 3.818 fold increased hazard ratio for death, p = 0.0001 (logrank test), n = 51.

SUPPLEMENTAL EXPERIMENTAL PROCEDURES

Polysome gradient analysis and qPCR. Cycloheximide (Sigma) was added for 3 minutes to the cells at 37°C. Two washes in ice-cold PBS containing cycloheximide were used to detach the cells from plates and then the cells were pelleted. Cell pellet were resuspended in ice cold lysis buffer (0.3M NaCl, 15mM MgCl2, 15mM Tris-HCl pH 7.5, 0.1mg/ml CHX, 1% Triton-X100). Lysates were centrifuged for 1 minute at 13 000rpm and the supernatant loaded onto a 10-50% sucrose gradient and subjected to ultracentrifugation at 38 000 rpm for 2 hours. Gradients were eluted using a Teledyne Isco density gradient fractionation system composed of UA-6 detector and optical unit, and a Foxy R1 fraction collector. RNAs were extracted from fractions using Trizol® (ThermoFisher Scientific) according to manufacturer's instructions. First strand cDNA synthesis was performed with equivalent volumes of extracted total RNA from each fraction was carried out using Quantitect® reverse transcription kit (Qiagen) according to manufacturer's instructions. For qPCR, cDNA was diluted 2-fold and 3 µl of this was used for analysis using PerfeCTa® SYBR® Green Fast Mix® (Quanta Biosciences). Quantitative PCR, carried out in a Bio-Rad CFX96, was performed thus; 95°C for 3 min followed by 40 cycles at 95°C for 20 s, 60°C for 20s and 72°C for 20s followed by a dissociation curve.

Isolation of primary cultured fibroblasts from breast cancer patients. Primary human breast fibroblasts were isolated from normal (reduction mammoplasty), tumour and surround (>5cm from tumour edge) tissue samples, following patient consent. Tissue was digested for 12 to 16 hours at 37˚C in RPMI-1640 medium plus 25mM Hepes, supplemented with 5% foetal bovine serum (FBS), penicillin (100U/ml), streptomycin (0.1mg/ml) and amphotericin-B (5µg/ml) containing 1mg/ml collagenase 1A and hyaluronidase on a rotary shaker. The digested tissue was centrifuged at 380g for 20 minutes and washed in medium three times to remove enzymes. The normal and surround tissue isolates were then sedimented three times at 1g for 30 mins to collect the denser organoids. The supernatants containing the fibroblasts were centrifuged (380g x 3minutes) and the cell pellets re-suspended and cultured in DMEM:F12 supplemented with 10% FBS and penicillin/streptomycin and amphotericin-B as above. Following centrifugation and washing steps, digested tumour tissue was filtered through a 40µm filter and the filtrate centrifuged and cultured as for the normal and surround cell isolates. Isolated cells were cultured for two to three passages prior to conducting experiments to guarantee the purity of the fibroblast populations.

Primer pairs used for qRT-PCR

Primer name	Primer Sequence 5' – 3'	Primer name	Primer Sequence 5' – 3'
$tRNA_i^{Met}$ Forward	AGAGTGGCGCAGCGGAAG	$tRNA_i^{Met}$ Reverse	AGCAGAGGATGGTTTCGATCC
$tRNA^{Ile}$ Forward	GGCGGCCGGTTAGCTCAG	$tRNA^{Ile}$ Reverse	CCCGTACGGGGATCGAAC
5S rRNA Forward	CAGCACCCGGTATTCCCAGG	5S rRNA Reverse	GGCATACCACCCTGAACGC
Collagen II Forward	GGGTCACAGAGGTTACCCAG	Collagen II Reverse	ACCAGGGGAACCACTCTCAC
ARPP P0 Forward	GCACTGGAAGTCCAACTACTTC	ARPP P0 Reverse	TGAGGTCCTCCTTGGTGAACAC
