# Supplementary material for: The Initiator Methionine tRNA Drives Secretion of Type II Collagen from Stromal Fibroblasts to Promote Tumor Growth and Angiogenesis
Source: Curr Biol. 2016 Mar 21;26(6):755–65. doi: 10.1016/j.cub.2016.01.045 (PMC4819511; doi:10.1016/j.cub.2016.01.045)
Supplement: Document S2. Article plus Supplemental Information [file mmc4.pdf]

Article

Current Biology

The Initiator Methionine tRNA Drives Secretion of Type II Collagen from Stromal Fibroblasts to Promote Tumor Growth and Angiogenesis

Highlights

- A mechanistic link between the tRNA repertoire and cancer progression
- Selective control of the secretome by levels of the initiator methionine tRNA
- A link between the tRNAome and ECM secretion from stromal fibroblasts
- A new transgenic model recapitulating a key aspect of the cancer tRNAome

Authors

Cassie J. Clarke, Tracy J. Berg, Joanna Birch, ..., Sara Zanivan, Andrew R. Reynolds, Jim C. Norman

Correspondence

andrew.reynolds@icr.ac.uk (A.R.R.), j.norman@beatson.gla.ac.uk (J.C.N.)

In Brief

Clarke et al. show that levels of the initiator $tRNA_i^{Met}$ are increased in carcinoma-associated fibroblasts. Using a transgenic approach to recapitulate this in vivo and quantitative mass spectrometry to characterize the stromal secretome, they show that elevated $tRNA_i^{Met}$ drives production of a type II collagen-rich ECM to drive tumor progression.

Clarke et al., 2016, Current Biology *26*, 755–765
March 21, 2016 ©2016 The Authors
http://dx.doi.org/10.1016/j.cub.2016.01.045

CrossMark

CellPress

Current Biology
Article

CellPress

The Initiator Methionine tRNA Drives Secretion of Type II Collagen from Stromal Fibroblasts to Promote Tumor Growth and Angiogenesis

Cassie J. Clarke,[1,7] Tracy J. Berg,[2,7] Joanna Birch,[1] Darren Ennis,[3] Louise Mitchell,[1] Catherine Cloix,[1] Andrew Campbell,[1] David Sumpton,[1] Colin Nixon,[1] Kirsteen Campbell,[1] Victoria L. Bridgeman,[2] Peter B. Vermeulen,[2,4] Shane Foo,[2] Eleftherios Kostaras,[2] J. Louise Jones,[5] Linda Haywood,[5] Ellie Pulleine,[6] Huabing Yin,[6] Douglas Strathdee,[1] Owen Sansom,[1] Karen Blyth,[1] Iain McNeish,[3] Sara Zanivan,[1] Andrew R. Reynolds,[2,*] and Jim C. Norman[1,*]
[1]Cancer Research UK Beatson Institute, Garscube Estate, Switchback Road, Glasgow G61 1BD, UK
[2]Tumour Biology Team, The Breast Cancer Now Toby Robins Research Centre, Mary-Jean Mitchell Green Building, The Institute of Cancer Research, London SW3 6JB, UK
[3]Wolfson Wohl Cancer Research Centre, Institute of Cancer Sciences, University of Glasgow, Glasgow G611QH, UK
[4]Translational Cancer Research Unit, GZA Hospitals St. Augustinus, Wilrijk 2610, Antwerp, Belgium
[5]Centre for Tumour Biology, Barts Cancer Institute, Queen Mary University of London, London EC1M 6BQ, UK
[6]School of Engineering, University of Glasgow, Glasgow G12 8LT, UK
[7]Co-first author
*Correspondence: andrew.reynolds@icr.ac.uk (A.R.R.), j.norman@beatson.gla.ac.uk (J.C.N.)
http://dx.doi.org/10.1016/j.cub.2016.01.045
This is an open access article under the CC BY license (http://creativecommons.org/licenses/by/4.0/).

SUMMARY

Expression of the initiator methionine tRNA ($tRNA_i^{Met}$) is deregulated in cancer. Despite this fact, it is not currently known how $tRNA_i^{Met}$ expression levels influence tumor progression. We have found that $tRNA_i^{Met}$ expression is increased in carcinoma-associated fibroblasts, implicating deregulated expression of $tRNA_i^{Met}$ in the tumor stroma as a possible contributor to tumor progression. To investigate how elevated stromal $tRNA_i^{Met}$ contributes to tumor progression, we generated a mouse expressing additional copies of the $tRNA_i^{Met}$ gene (2+$tRNA_i^{Met}$ mouse). Growth and vascularization of subcutaneous tumor allografts was enhanced in 2+$tRNA_i^{Met}$ mice compared with wild-type littermate controls. Extracellular matrix (ECM) deposited by fibroblasts from 2+$tRNA_i^{Met}$ mice supported enhanced endothelial cell and fibroblast migration. SILAC mass spectrometry indicated that elevated expression of $tRNA_i^{Met}$ significantly increased synthesis and secretion of certain types of collagen, in particular type II collagen. Suppression of type II collagen opposed the ability of $tRNA_i^{Met}$-overexpressing fibroblasts to deposit pro-migratory ECM. We used the prolyl hydroxylase inhibitor ethyl-3,4-dihydroxybenzoate (DHB) to determine whether collagen synthesis contributes to the $tRNA_i^{Met}$-driven pro-tumorigenic stroma in vivo. DHB had no effect on the growth of syngeneic allografts in wild-type mice but opposed the ability of 2+$tRNA_i^{Met}$ mice to support increased angiogenesis and tumor growth. Finally, collagen II expression predicts poor prognosis in high-grade serous ovarian carcinoma. Taken together, these data indicate that increased $tRNA_i^{Met}$ levels contribute to tumor progression by enhancing the ability of stromal fibroblasts to synthesize and secrete a type II collagen-rich ECM that supports endothelial cell migration and angiogenesis.

INTRODUCTION

The use of transcriptomics and proteomics to understand cellular function has been pivotal in advancing our knowledge of the molecular basis of disease processes, and the combined use of these approaches is beginning to highlight discord between the control of gene expression at the levels of transcription and translation. Indeed, implementation of numerous post-transcriptional processes means that expression of particular genes at the mRNA level does not necessarily correlate with cellular concentrations of functional products of the same transcripts. tRNAs are one of the most-abundant RNAs in eukaryotic cells, yet are frequently disregarded in most genome-wide analyses because they are assumed to be background genes with merely housekeeping functions. However, it is now becoming clear that cellular levels of tRNAs are key to the control of gene expression in a number of different contexts. Indeed, tissue-specific differences in tRNA expression occur across a variety of human tissues, with relative levels of tRNAs correlating to the codon usage of highly expressed genes in those tissues [1]. Indeed, the availability of certain tRNAs may be controlled in the cell in order to regulate the efficiency of translation of certain gene transcripts. This may, in turn, allow the cell to regulate the activity of certain pathways [2]. For instance, proliferating cells have increased levels of tRNAs that are specific for codons overrepresented in pathways required for proliferation. A number of publications have demonstrated that tRNA levels may be increased in cancer [3–5], and although it was previously unknown whether this was a cause or consequence of carcinogenesis, recent work from

CrossMark

Gingold et al. [2] highlighted the possibility that tRNA expression could indeed play a role in carcinogenesis itself.

There has also been some focus on the consequences of altered expression of specific tRNAs, including $tRNA_i^{Met}$, the initiator methionine tRNA responsible for recognizing the start codon and initiating translation. Whereas one group indicated that overexpression of $tRNA_i^{Met}$ may reprogram global tRNA expression levels and increase proliferation of human epithelial cells [6], another showed that increased levels of $tRNA_i^{Met}$ influences the growth of *Drosophila* larvae largely owing to increased secretion of insulin-related peptides from fat bodies [7]. This in vivo observation that $tRNA_i^{Met}$ levels can drive protein secretion is particularly interesting. It indicates that $tRNA_i^{Met}$-sensitive translational control may contribute to cancer progression not only by altering gene expression of the cancer cells themselves but also by altering the secretome of other cells that support carcinogenesis [8–10].

In the current study, we find that levels of $tRNA_i^{Met}$ are increased in cancer-associated fibroblasts (CAFs) by comparison to normal fibroblasts. To further understand the consequences of altered stromal expression of $tRNA_i^{Met}$, we generated a transgenic mouse that carries two additional copies of the $tRNA_i^{Met}$ gene (2+$tRNA_i^{Met}$ mouse). We found that this leads to enhanced tumor growth and enhanced tumor vascularization. In terms of mechanism, we show that increased tumor growth in 2+$tRNA_i^{Met}$ mice is due to elevated secretion of type II collagen by fibroblasts which, in turn, supports migration of endothelial cells, leading to increased tumor vascularity. Our data therefore provide the first evidence that $tRNA_i^{Met}$ levels selectively drive secretion of specific components of the extracellular matrix (ECM) to support tumor progression.

RESULTS

Increased Expression of $tRNA_i^{Met}$ in the Host Animal Promotes Tumor Growth and Angiogenesis

To determine whether tRNA expression is altered in the tumor stroma, we measured tRNA levels in immortalized human mammary carcinoma-associated fibroblasts (iCAFs) and compared these with immortalized matched normal fibroblast (iNF) controls. iCAFs were generated by co-injecting iNFs with Ras-transformed MCF7 cells subcutaneously into mice [11]. These iCAFs are established to have an enhanced capacity to promote tumor growth and angiogenesis when co-injected as xenografts with a variety of tumor cell types and are established to maintain their myofibroblast-like phenotype when grown in culture [11]. We characterized these cells for the expression of non-coding RNA transcribed by RNA polymerase III. Importantly, increased expression of $tRNA_i^{Met}$ and the tRNA for isoleucine ($tRNA^{Ile}$) was seen in iCAFs by comparison with iNFs, whereas no change was detected in the expression of the 5S rRNA, another non-coding RNA transcribed by RNA polymerase III (Figure S1).

To investigate the consequences of increased stromal $tRNA_i^{Met}$, we generated a transgenic mouse that carries two extra copies of the $tRNA_i^{Met}$ gene inserted into the genome at the HPRT locus (2+$tRNA_i^{Met}$ mouse; Figure S2A). 2+$tRNA_i^{Met}$ animals have elevated levels (1.3- to 1.5-fold) of $tRNA_i^{Met}$ in a number of tissues, including fibroblasts from lung and from whole-mouse embryos (MEFs) (Figure S2B). 2+$tRNA_i^{Met}$ animals were the same size and weight as their littermate controls (not shown), and MEFs isolated from 2+$tRNA_i^{Met}$ embryos did not have altered rates of cellular protein synthesis (measured by ^{35}S-methionine incorporation) or cell proliferation (Figures S2C and S2D). We introduced syngeneic tumor cells into 2+$tRNA_i^{Met}$ mice as subcutaneous allografts and recorded their growth. Initially, we used transformed melanoblasts derived from Tyr::NrasQ61K/°;INK4a$^{-/-}$ mice. These transformed melanoblasts are null for INK4a, express mutant NRas under a melanoblast-specific promoter, and form melanomas in syngeneic host mice [12]. Mice were culled once tumors reached a specified size (15 × 15 mm), which we defined as the clinical endpoint in order to perform Kaplan-Meier survival analysis. Importantly, tumors reached clinical endpoint significantly faster in 2+$tRNA_i^{Met}$ mice than they did in wild-type littermate controls (Figure 1A). To confirm this observation of enhanced tumor growth in 2+$tRNA_i^{Met}$ mice, we also measured the growth of Lewis lung carcinoma (LLC) and B16 F0 melanoma allografts. Both allograft models formed significantly larger and more-vascularized tumors in 2+$tRNA_i^{Met}$ mice than they did when grown in wild-type littermate control animals (Figures 1B and 1C), indicating that increased $tRNA_i^{Met}$ expression in the host animal can promote increased tumor growth and tumor angiogenesis.

Increased Stromal $tRNA_i^{Met}$ Expression Promotes the Deposition of a Pro-migratory ECM via Release of Secreted Factors

A key component of the tumor microenvironment that dictates cancer cell behavior is the ECM deposited by stromal fibroblasts. To determine whether $tRNA_i^{Met}$ levels influence the characteristics of fibroblast-derived ECM, we isolated fibroblasts from the lungs and embryos of 2+$tRNA_i^{Met}$ mice (Figure S2B) and used these to prepare cell-derived matrices using established methods [13, 14]. We then investigated the capacity of this cell-free ECM to support the migration of other cells. ECM deposited by primary cultured fibroblasts from 2+$tRNA_i^{Met}$ mice had significantly enhanced ability to support migration of both endothelial cells (Figure 2A) and fibroblasts (Figure 2B) by comparison with ECM deposited by cells from their wild-type littermate controls. As a tool to determine how increased $tRNA_i^{Met}$ expression influences ECM deposition, we generated independent pools of immortalized mouse embryonic fibroblasts (iMEF) transfected with either $tRNA_i^{Met}$ (iMEF-$tRNA_i^{Met}$) or an empty vector control (iMEF-vector) (Figure S3A). Consistent with our observations in primary cultured MEFs, increased $tRNA_i^{Met}$ expression did not lead to increased cell proliferation or cellular protein synthesis in immortalized MEFs (Figures S3B and S3C). However, ECM deposited by iMEF-$tRNA_i^{Met}$ cells had a similarly enhanced ability to support increased cell migration as did the ECM secreted by primary cultured fibroblasts from 2+$tRNA_i^{Met}$ mice (Figure 3A).

To determine whether the relationship between $tRNA_i^{Met}$ expression and the deposition of pro-migratory ECM was observable in fibroblasts from human tumors, we obtained primary CAFs from two human breast tumors and compared these with fibroblasts isolated from the surrounding normal tissue (>5 cm from the tumor) from the same patients. The CAFs from patient 1 displayed significant upregulation of $tRNA_i^{Met}$ by comparison with fibroblasts from surrounding normal tissue,

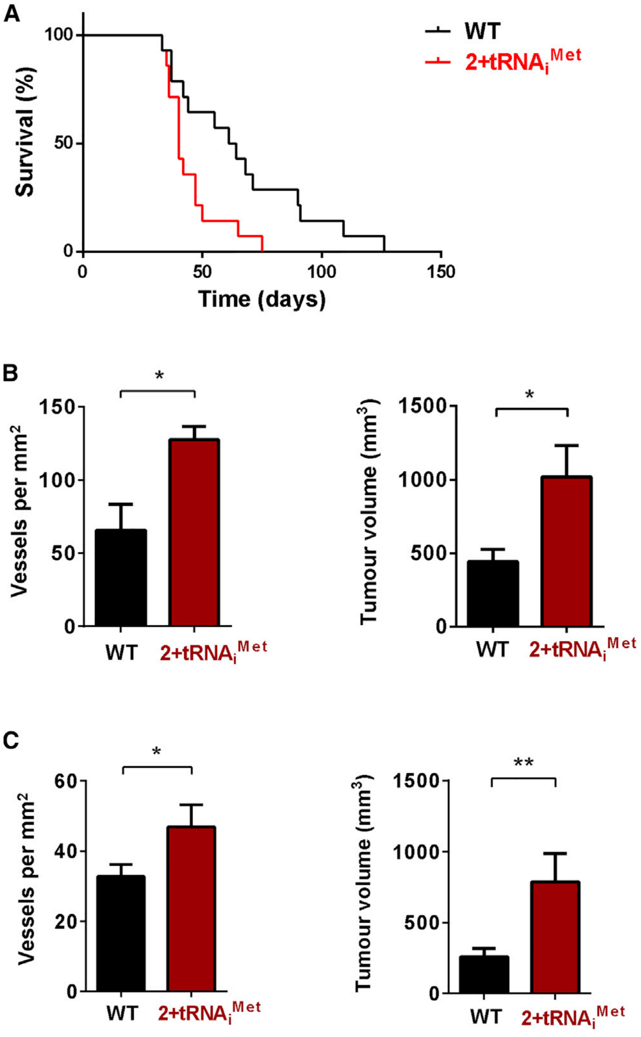

Figure 1. Increased Expression of tRNA$_i^{Met}$ in the Host Animal Promotes Angiogenesis and Growth of Allografts

(A) 1 × 10^6 transformed melanocytes (Tyr::NrasQ61K/°;INK4a−/−) were injected subcutaneously into either wild-type (WT) or 2+tRNA$_i^{Met}$ transgenic male mice and tumor size monitored. Mice were culled once tumors reached endpoint (15 × 15 mm in size). Kaplan-Meier survival curves were plotted based on time taken to reach endpoint. n = 14 (WT); n = 14 (2+tRNA$_i^{Met}$ mice); log rank (Mantel-Cox) test; *$p < 0.05$.

(B) 1 × 10^6 LLC cells were injected subcutaneously into either wild-type (WT) or 2+tRNA$_i^{Met}$ transgenic male mice, and tumors were harvested at 21 days. The graph on the right shows final tumor volume ± SEM. Tumors were fixed and stained to visualize endomucin for blood vessel identification and vessels counted across the entire tumor section. Graph on the left shows tumor vessel density ± SEM; n = 10 (WT); n = 10 (2+tRNA$_i^{Met}$); unpaired t test; *$p < 0.05$.

(C) 0.5 × 10^6 B16 F0 mouse melanoma cells were injected subcutaneously into either wild-type (WT) or 2+tRNA$_i^{Met}$ transgenic mice. The time at which palpable tumors were formed was recorded (minimum size of 3 × 3 mm) and then tumors were harvested 22 days from this time point. Tumors were fixed and stained to visualize endomucin for blood vessel identification and vessels counted across the entire tumor section. The graph on the right shows final tumor volume ± SEM. The graph on the left shows tumor vessel density ± SEM; n = 20 (WT); n = 20 (2+tRNA$_i^{Met}$); unpaired t test; *$p < 0.05$; **$p < 0.005$.

but this was not observed in the second patient (patient 2) (Figure S4A). Interestingly, the ECM produced by CAFs from patient 1 supported significantly increased cell migration, whereas the CAF-derived ECM from patient 2 did not (Figure S4B). These data indicate that, although upregulation of tRNA$_i^{Met}$ in CAFs varies between patients, the relationship between tRNA$_i^{Met}$ levels and the ability to generate pro-migratory ECM may be observed in primary cultured CAFs from human tumors.

Many ECM components are initially secreted by fibroblasts as soluble proteins that are subsequently incorporated into the insoluble ECM. To determine which of these stages of ECM deposition are influenced by tRNA$_i^{Met}$ levels, we collected conditioned medium from iMEF-tRNA$_i^{Met}$ and iMEF-vector cells and incubated these with normal iMEFs, which were, in turn, allowed to deposit ECM. Interestingly, ECM deposited by iMEFs cultured in conditioned medium from tRNA$_i^{Met}$-overexpressing cells had significantly enhanced ability to support cell migration (Figure 3B), indicating that tRNA$_i^{Met}$ levels control the synthesis and secretion of soluble ECM components rather than the ability of fibroblasts to remodel these and incorporate them into insoluble ECM.

Cell migration may be influenced by the mechanical properties of the ECM. ECM stiffness promotes cell migration, and certain secreted enzymes (such as lysyl oxidases and transglutaminases) catalyze the formation of covalent cross-links between amino acid chains to increase ECM stiffness. We used atomic force microscopy (AFM) to measure the mechanical properties of ECM and found that cell-derived matrix generated in the presence of conditioned medium from tRNA$_i^{Met}$-overexpressing cells did not consistently differ in its thickness or stiffness from that generated in the presence of conditioned medium from control cells (Figure 3C). Taken together, these data indicate that increased tRNA$_i^{Met}$ expression supports deposition of a pro-migratory ECM via release of secreted components that do not influence the mechanical properties of the ECM.

Increased tRNA$_i^{Met}$ Levels Drive a Secretome that Is Enriched in Type II Collagen

We used SILAC-based mass spectrometry to quantitatively determine the influence of tRNA$_i^{Met}$ levels on the complement of soluble proteins secreted by fibroblasts. We labeled iMEF-tRNA$_i^{Met}$ and iMEF-vector cells with light and heavy SILAC amino acids, respectively (forward [Fw] labeling), and with heavy and light amino acids, respectively (reverse [Rev] labeling). We then prepared cell extracts, and for the secretome, we used an affinity bead (Strataclean)-based approach to enrich for proteins secreted into serum-free conditioned medium [15]. Proteomes were detected using an LTQ-Orbitrap, and mass spectrometry data were analyzed with the MaxQuant computational platform, which identified proteins in the proteome and in the secretome (Tables S1 and S2) with high correlation between Fw and Rev experiments. Analysis of the cellular proteome indicated that increased tRNA$_i^{Met}$ levels disproportionately increased the synthesis of proteins destined for secretion (Figure 4A; Table S1) whereas not greatly affecting the production of cellular non-secreted components. By contrast, the composition of the secretome was significantly altered by increased expression of tRNA$_i^{Met}$ (Figures 4B and 4C). Indeed, tRNA$_i^{Met}$ overexpression lead to enhanced production of many secreted ECM components, including collagens and collagen-processing enzymes

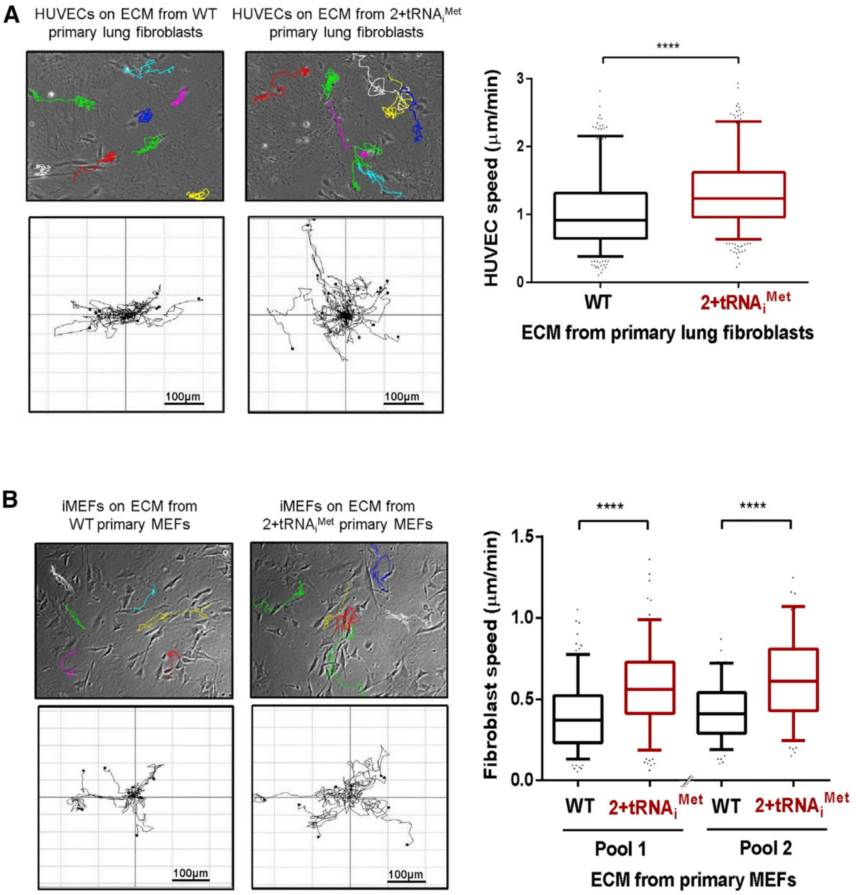

Figure 2. Fibroblasts Isolated from 2+tRNA$_i^{Met}$ Transgenic Mice Deposit a Pro-migratory ECM

(A) Extracellular matrix (ECM) was generated from primary lung fibroblasts isolated from either wild-type (WT) or 2+tRNA$_i^{Met}$ transgenic mice. See Figure S2 for characterization of these cells. Migration of human umbilical vein endothelial cells (HUVECs) on the ECM was recorded by time-lapse microscopy and analyzed using ImageJ. Data represent ECM generated from seven independent isolates of primary fibroblasts from pools of three WT and three 2+tRNA$_i^{Met}$ transgenic mice, tracking the migration of at least 28 HUVECs in each replicate. Box and whisker plot represents 5–95 percentile; Mann-Whitney test; ****$p < 0.0001$.

(B) ECM was generated from primary fibroblasts isolated from either WT or 2+tRNA$_i^{Met}$ primary mouse embryonic fibroblasts (MEFs)—these cells are characterized in Figure S2. Migration of immortalized MEFs (iMEFs) on the ECM was recorded by time-lapse microscopy over a 17-hr time course and analyzed using ImageJ. Data represent ECM generated from primary fibroblasts from two pairs of litter-matched WT and 2+tRNA$_i^{Met}$ transgenic mice, tracking the migration of at least 60 iMEFs in each replicate; n = 3 for pair 1; n = 2 for pair 2. Box and whisker plot represents 5–95 percentile; Mann-Whitney test; ****$p < 0.0001$.

(Figure 4C). In particular, type II collagen secretion by fibroblasts was upregulated by at least 50-fold following tRNA$_i^{Met}$ overexpression in these cells (Figures 4B and 4C; Table S2). Furthermore, specificity in the nature of the secreted ECM components was observed in iMEF-tRNA$_i^{Met}$ cells, which did not necessarily correlate with protein abundance (Figure 4D). For example, despite the observation that fibronectin was one of the most-abundant proteins in the secretome, its levels were not significantly altered following tRNA$_i^{Met}$ overexpression (Figures 4C and 4D). Upregulated expression of a given protein is commonly associated with increased recruitment of its mRNA(s) to actively translating ribosomes (polysomes). We used sucrose density gradients to determine whether increased tRNA$_i^{Met}$ levels were able to drive polysomal recruitment of collagen II mRNA. We found no indication that the monosome/polysome ratio differed between iMEF-vector and iMEF-tRNA$_i^{Met}$ cells (Figure S5A), which is consistent with our observations that the overall levels of cellular protein synthesis were unaffected by increased tRNA$_i^{Met}$ levels (Figures S2C and S3C). However, iMEF.tRNA$_i^{Met}$ cells displayed markedly increased polysomal content of collagen II mRNA (Figure S5B). Conversely, polysomal association of mRNAs for fibronectin and the ARPP0 ribosomal protein with polysomes was unaffected by increased tRNA$_i^{Met}$ levels (Figures S5C and S5D).

Immunostaining of ECM deposited by two independent tRNA$_i^{Met}$-overexpressing cell lines confirmed that upregulation of soluble secreted type II collagen was also reflected by its increased incorporation into the insoluble ECM (Figure 4E). We examined whether levels of tRNA$_i^{Met}$ in the stroma influences the collagen II content of tumors in vivo. To do this, we introduced LLC cells into wild-type and 2+tRNA$_i^{Met}$ mice as syngeneic allografts, allowed the tumors to grow for 21 days, and then stained them for collagen II. Collagen II deposition was clearly visible in the spaces between tumor cells, and quantitative analysis indicated that the collagen II content of tumors grown in 2+tRNA$_i^{Met}$ animals was significantly higher than it was in tumors from littermate control animals (Figures 5A and 5B).

Type II Collagen Secretion Is Required for tRNA$_i^{Met}$ to Drive Production of Pro-tumorigenic ECM

To assess whether type II collagen secretion is responsible for the ability of tRNA$_i^{Met}$ to deposit a pro-migratory ECM, we generated cell-derived matrices from iMEFs cultured with conditioned media from iMEF-tRNA$_i^{Met}$ cells transfected with type II collagen-specific siRNA or non-targeting control. Knockdown of type II collagen in iMEF-tRNA$_i^{Met}$ cells resulted in conditioned medium with decreased ability to enable iMEFs to generate ECM that supported fibroblast migration (Figures 6A and S6A–S6C). Furthermore, knockdown of type II collagen (with two independent siRNA sequences) in primary lung fibroblasts isolated from 2+tRNA$_i^{Met}$ mice similarly compromised the ability of the ECM derived from these cells to support increased migration of endothelial cells (Figure 6B).

ECM was also made from iMEFs in which type II collagen levels had been suppressed using CRISPR genome editing (MEF-tRNA$_i^{Met}$/collagen II CRISPR; Figures S6D and S6E). Immunostaining indicated significantly decreased type II collagen

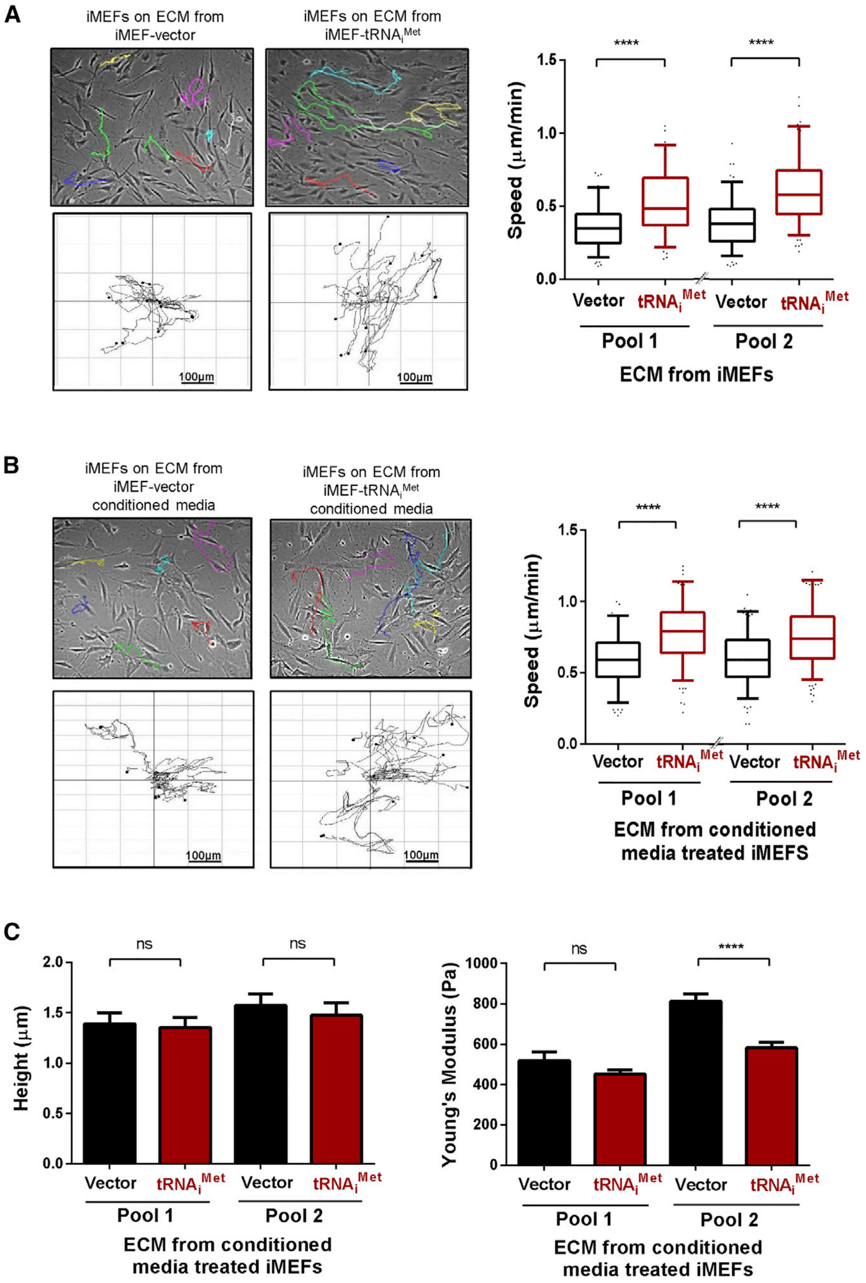

Figure 3. tRNA$_i^{Met}$ Supports Deposition of Pro-migratory ECM via Release of Secreted Factors

(A) Migration of iMEFs on ECM was recorded by time-lapse microscopy over a 17-hr period and analyzed using ImageJ. ECM was generated using immortalized fibroblasts (iMEFs) stably overexpressing either empty vector (iMEF-vector) or tRNA$_i^{Met}$ (iMEF-tRNA$_i^{Met}$). See Figure S3 for characterization of these cells.

(B) ECM was generated by iMEFs in the presence of conditioned media from iMEF-vector or iMEF-tRNA$_i^{Met}$ cells. All data represent ECM generated from at least three independent ECM isolations, tracking the migration of at least 40 iMEFs in each replicate. Box and whisker plot represents 5–95 percentile; Mann-Whitney test; ****$p < 0.0001$.

(C) Atomic force microscopy and contact imaging were performed using a JPK NanoWizard II Bio AFM in combination with a Bruker MLCT cantilever and to measure the thickness of ECM following wound scratch or a Nanoworld Arrow TL-1 cantilever with bead attached for force spectroscopy; n = 3 independent ECM generations; ±SEM; ****$p < 0.0001$; ns, no significant difference; Kruskal-Wallis test.

incorporation into ECM deposited by MEF-tRNA$_i^{Met}$/collagen II CRISPR cells (Figure 6C, left panel). Consistently, ECM generated by MEF-tRNA$_i^{Met}$/collagen II CRISPR fibroblasts had decreased ability to support increased cell migration (Figures 6C, right panel, and S6F).

We then determined whether collagen synthesis was required for generation of a pro-tumorigenic microenvironment in vivo. To do this, we used ethyl-3,4-dihydroxybenzoate (DHB), a prolyl hydroxylase inhibitor that is well-established to compromise collagen processing in vivo and thus prevents collagen secretion in vivo [16]. Indeed, DHB administration significantly reduced the collagen II content of allografted tumors in both wild-type and tRNA$_i^{Met}$ mice (Figures 5A and 5B). However, whereas DHB treatment did not affect vascularity or growth of LLC subcutaneous allografts in WT mice, it significantly opposed the increased vascularity and growth of tumors seen in 2+tRNA$_i^{Met}$ mice (Figure 5C), indicating that collagen production is indeed required for tRNA$_i^{Met}$ to drive tumor growth and angiogenesis in vivo.

Type II Collagen Expression Predicts Poor Prognosis in Ovarian Cancer

To determine whether the relationship between increased collagen II levels and tumor progression is observable in human cancer, we stained a tissue microarray containing 62 cases of primary ovarian tumors. The decision to focus on this cancer type was influenced by reports that ovarian cancer aggressiveness is particularly associated with increased angiogenesis [17]. In univariate analysis, high collagen II histoscores were associated with significantly decreased survival following resection of primary ovarian tumors (Figure 7), and this was particularly apparent in high-grade serous carcinoma (Figure S7). Indeed, patients with tumors displaying a collagen II histoscore greater than 50 have a 2.992-fold increased hazard ratio for death ($p = 0.0002$). These data indicate that collagen II expression is a significant factor in driving the progression of ovarian cancer.

DISCUSSION

Regulation of the cellular tRNA repertoire is thought to dictate selectivity of translational programs to influence cancer progression and to define cell fates [2]. Indeed, the tRNA repertoire

CellPress

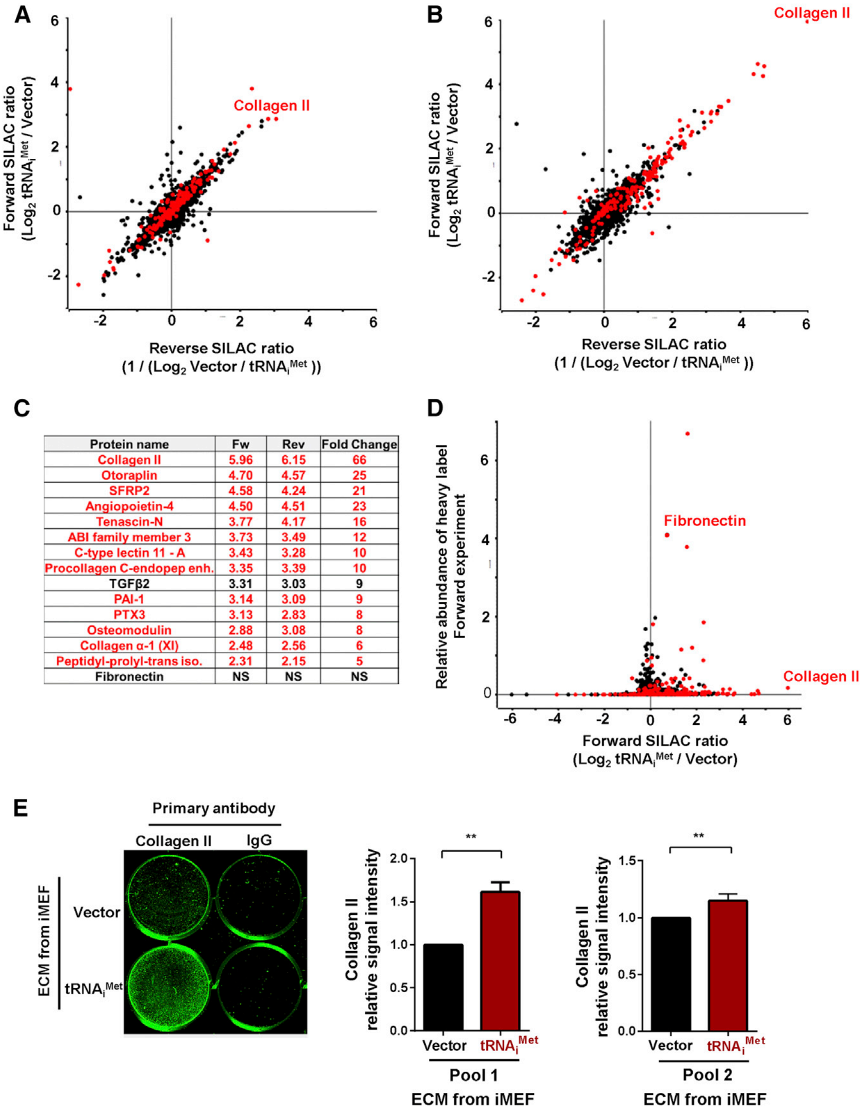

Protein name	Fw	Rev	Fold Change
Collagen II	5.96	6.15	66
Otoraplin	4.70	4.57	25
SFRP2	4.58	4.24	21
Angiopoietin-4	4.50	4.51	23
Tenascin-N	3.77	4.17	16
ABI family member 3	3.73	3.49	12
C-type lectin 11 - A	3.43	3.28	10
Procollagen C-endopep enh.	3.35	3.39	10
TGFβ2	3.31	3.03	9
PAI-1	3.14	3.09	9
PTX3	3.13	2.83	8
Osteomodulin	2.88	3.08	8
Collagen α-1 (XI)	2.48	2.56	6
Peptidyl-prolyl-trans iso.	2.31	2.15	5
Fibronectin	NS	NS	NS

Figure 4. $tRNA_i^{Met}$ Drives a Secretome that Is Enriched in Type II Collagen

(A) The cellular proteome was analyzed following $tRNA_i^{Met}$ overexpression using quantitative SILAC mass spectrometry. iMEF-vector and iMEF-$tRNA_i^{Met}$ cells were labeled with heavy and light amino acids. Cells were lysed and proteins separated by gel electrophoresis and analyzed by mass spectrometry. Forward experiment (Fw) corresponds to iMEF-$tRNA_i^{Met}$ labeled with heavy amino acids combined with iMEF-vector light amino acids, and the reverse experiment (Rev) corresponds to iMEF-vector heavy-labeled amino acids combined with iMEF-$tRNA_i^{Met}$ light amino acids. ECM proteins are highlighted in red.

(B) The secretome was analyzed following $tRNA_i^{Met}$ overexpression using quantitative SILAC mass spectrometry. Cells were labeled as for (A), and conditioned medium was collected. Proteins were concentrated from conditioned medium using Strataclean beads and subjected to mass spectrometry analysis as for (A). ECM proteins are highlighted in red.

(C) A table of the most significantly upregulated secreted proteins identified in conditioned medium following $tRNA_i^{Met}$ overexpression. Upregulated ECM proteins are highlighted in red.

(D) Data from (B) replotted to show the total abundance of secreted proteins identified in the media of iMEF-$tRNA_i^{Met}$ against the fold change in expression of the same proteins in the iMEF-$tRNA_i^{Met}$ compared to iMEF-vector. ECM proteins are highlighted in red.

(E) ECM was generated from iMEF-vector and iMEF-$tRNA_i^{Met}$ cells. Incorporation of collagen II into the ECM was assessed by immunostaining using type II collagen-specific antibodies and quantified using the Aerius infrared imaging system (LI-COR Biosciences).

differs between proliferating and differentiated cells, and this is thought to influence the translational landscape according to codon usage [2, 18]. Thus, an mRNA with a preponderance of a given codon will be translated more rapidly if the tRNA recognizing that codon is abundant. Consequently, one might anticipate that increased $tRNA_i^{Met}$ (which recognizes AUG at translation start sites) would generally increase translation initiation and globally upregulate protein synthesis and cell growth [6]. However, our transgenic approach has given no indications that increased $tRNA_i^{Met}$ levels promote cell growth or proliferation in a cell-autonomous fashion, nor does this greatly influence the quantity or profile of cellular protein synthesis. Instead, we have found that increased levels of $tRNA_i^{Met}$ lead to highly selective adjustments to the protein synthesis landscape that are restricted to certain secreted proteins—particularly the ECM component, type II collagen. There is evidence from studies in *Drosophila* that elevated $tRNA_i^{Met}$ controls production of secreted proteins. Introduction of one extra copy of the $tRNA_i^{Met}$ gene into flies is sufficient to markedly enhance larval growth [7]. Consistent with our findings, this is primarily not attributable to a cell-autonomous drive to cell growth and proliferation in the developing organs. Rather, growth is driven by an endocrine relay in which increased $tRNA_i^{Met}$ in fat body cells promotes secretion of factor(s) that trigger release of insulin-like peptides from brain neurosecretory cells that, in turn, circulate throughout the larvae to promote growth of developing organs and tissues [7].

ECM production constitutes a significant commitment to the cell (both in terms of load on the ER and the biosynthetic machinery), indicating the need for special mechanisms to be in place to control the translation of ECM proteins and other abundant components of the fibroblast secretome. Expression of type I collagens is known to be controlled at the level of mRNA stability and translation, due to unique 5′ stem loops in their 5′ UTRs [19], and the production of these fibrillar collagens by the stroma is established to influence cancer growth and metastasis [20, 21]. Type II collagen is also a fibrillar collagen; however, little is known about how its production might be controlled post-transcriptionally. Although the sensitivity of collagen II production to $tRNA_i^{Met}$ levels indicates that translation initiation is likely a key point of

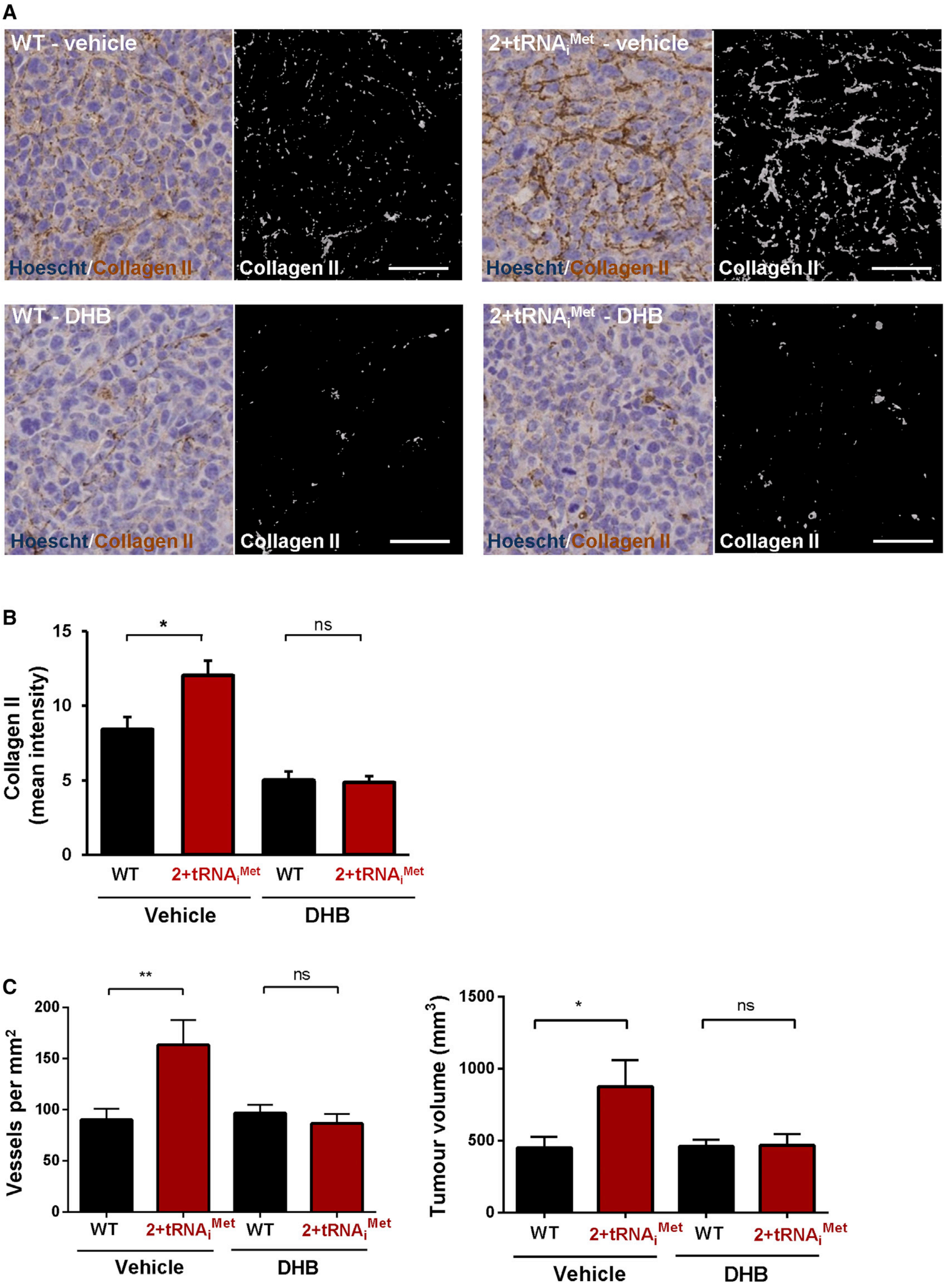

Figure 5. Collagen II Production Is Required for tRNA$_i^{Met}$ to Drive Production of a Pro-tumorigenic ECM

1 × 10^6 LLC cells were injected subcutaneously into either WT or 2+tRNA$_i^{Met}$ transgenic male mice, followed by daily administration of ethyl-3,4-dihydroxybenzoate (DHB) (40 mg/kg) or vehicle control. Tumors were harvested 21 days following injection. Tumor volumes were measured and tumor sections were stained for collagen II or endomucin.

(A and B) Collagen II staining in LLC tumors harvested from WT and 2+tRNA$_i^{Met}$ mice treated with vehicle or DHB as indicated (A). Collagen II staining was quantified by using the Image J "threshold color" plug-in to highlight the brown staining in the left panels and to render this as a grayscale image as shown in

(legend continued on next page)

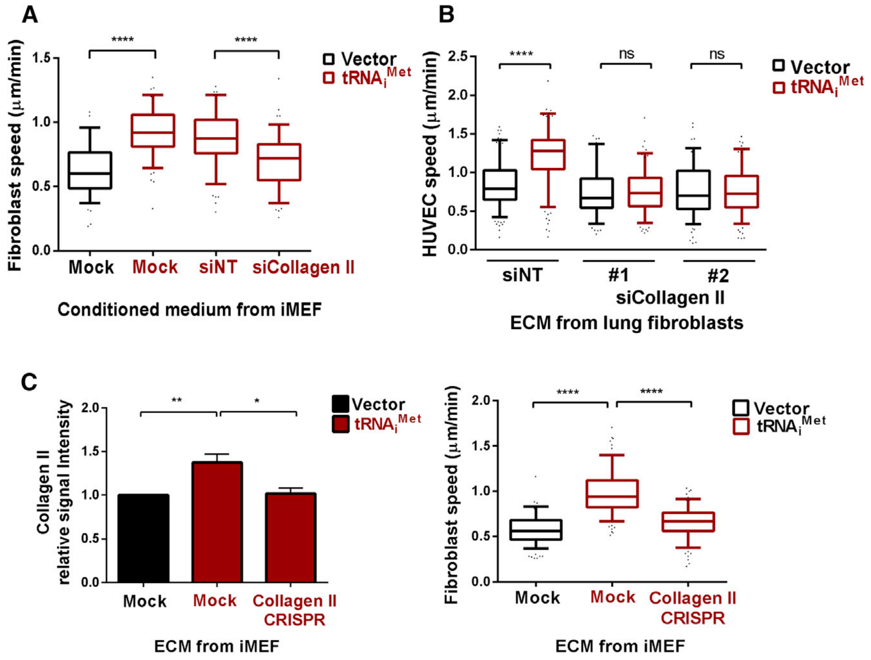

Figure 6. Collagen II Secretion Is Required for tRNA$_i^{Met}$ to Drive Production of a Pro-migratory ECM

(A) ECM was generated from iMEFs in the presence of conditioned media from iMEF-tRNA$_i^{Met}$ cells treated with non-targeting (siNT) or type-II-collagen-specific siRNA (siCollagen II). Migration of immortalized MEFs (iMEFs) on the ECM was recorded by time-lapse microscopy over a 17-hr time course and analyzed using ImageJ. See Figures S6A–S6C for a schematic representation of the protocol for this experiment, the validation of the siRNA, and representative examples of the migration track plots.

(B) ECM was generated from primary lung fibroblasts treated with non-targeting (siNT) or type-II-collagen-specific siRNA (siCollagen II). Two independent pools of fibroblasts from 2+tRNA$_i^{Met}$ mice were used as indicated. Migration of human umbilical vein endothelial cells (HUVECs) on the ECM was recorded by time-lapse microscopy and analyzed using ImageJ.

(C) ECM was generated from iMEF-vector, iMEF-tRNA$_i^{Met}$, and iMEF-tRNA$_i^{Met}$ with type II collagen II stable knockdown (iMEF-tRNA$_i^{Met}$ Collagen II CRISPR). Incorporation of collagen II into the ECM was assessed by immunostaining using type-II-collagen-specific antibodies, quantified using the Aerius infrared imaging system (LI-COR Biosciences); unpaired t test; *$p < 0.05$; **$p < 0.005$. Migration of iMEFs was recorded as previously described. Data represent ECM generated from at least three independent ECM isolations, tracking the migration of at least 40 iMEFs in each replicate. Box and whisker plot represents 5–95 percentile; Kruskal-Wallis test; ****$p < 0.0001$; ns, not significant. See Figures S6D–S6F for a schematic representation of the protocol for this experiment, the validation of the CRISPR, and representative examples of the migration track plots.

control of type II collagen synthesis, there are indications that the mechanism for this might not be straightforward. Indeed, in some cells (including those used for the polysome analysis displayed in Figure S5), we find that tRNA$_i^{Met}$ overexpression increases the cellular levels of mRNA for type II collagen (not shown), indicating that tRNA$_i^{Met}$ levels may influence transcription or stability of mRNAs for ECM proteins. Thus, despite clear indications that tRNA$_i^{Met}$ levels selectively control ECM protein synthesis in a way that influences tumor outcomes, further work will be needed to fully establish the molecular mechanisms through which tRNA$_i^{Met}$ levels influence type II collagen synthesis and the contribution made by the translational machinery to this non-cell-autonomous drive to tumor progression.

Despite the presence of a number of tRNA$_i^{Met}$-sensitive components in the fibroblast secretome, it is clear that suppression of just one of these, type II collagen, is sufficient to negate the tRNA$_i^{Met}$ phenotype. Moreover, we have shown that stromal levels of type II collagen are strongly associated with aggressiveness of serous ovarian carcinoma, indicating the importance of this tRNA$_i^{Met}$-sensitive secreted factor to the progression of a human cancer type. However, we have been unable to quantitatively determine tRNA$_i^{Met}$ levels in human tumor microarrays, and it will be necessary to do this to conclusively determine whether stromal tRNA$_i^{Met}$ drives type II collagen synthesis in human tumors in the same way as we have demonstrated by performing allografts in our 2+tRNA$_i^{Met}$ mouse model. Type II collagen is an abundant component of cartilage and is produced in large quantities by chondrocytes. By contrast, normal fibroblasts are not thought to secrete collagen II, and consistently, our SILAC data show that this collagen is a relatively minor component of the fibroblast secretome. Thus, acquisition of type II collagen secretion when tRNA$_i^{Met}$ levels are increased may represent a shift to a more-chondrocyte-like phenotype, and this may be consistent with the role for the tRNA repertoire in influencing differentiation state [2]. In view of this, one might anticipate that increased tRNA$_i^{Met}$ levels may be associated with production of ECM with cartilaginous features, such as increased stiffness. However, our AFM data show clearly that ECM produced by fibroblasts with increased tRNA$_i^{Met}$ levels does not have consistently altered compliance, indicating that type II collagen content is not necessarily linked to stiffness. In addition to providing mechanical support, a key role of the ECM is to act as a reservoir for growth factors and other bioactive proteins. The tRNA$_i^{Met}$ secretome contains a number of pro-tumorigenic growth factors (such as frizzled-related protein and TGF-β2), and we are currently considering the possibility that type II collagen production is associated with secretion of these growth factors and their incorporation into the ECM, increasing their presentation to tumor and endothelial cells.

the right-hand panels. Bar, 50 μm. Graph in (B) shows quantification of collagen II staining in LLC tumors harvested from WT and 2+tRNA$_i^{Met}$ mice treated with vehicle or DHB.

(C) Graphs show tumor volumes (right-hand graph) and quantification of endomucin-positive tumor blood vessel density (left-hand graph) in harvested tumors from WT and 2+tRNA$_i^{Met}$ mice treated with vehicle or DHB as indicated. Values are mean ± SEM; n = 8 (WT; vehicle); n = 7 (2+tRNA$_i^{Met}$; vehicle); n = 8 (WT; DHB); n = 7 (2+tRNA$_i^{Met}$; DHB); unpaired t test; *$p < 0.05$; **$p < 0.001$; ns, no significant difference.

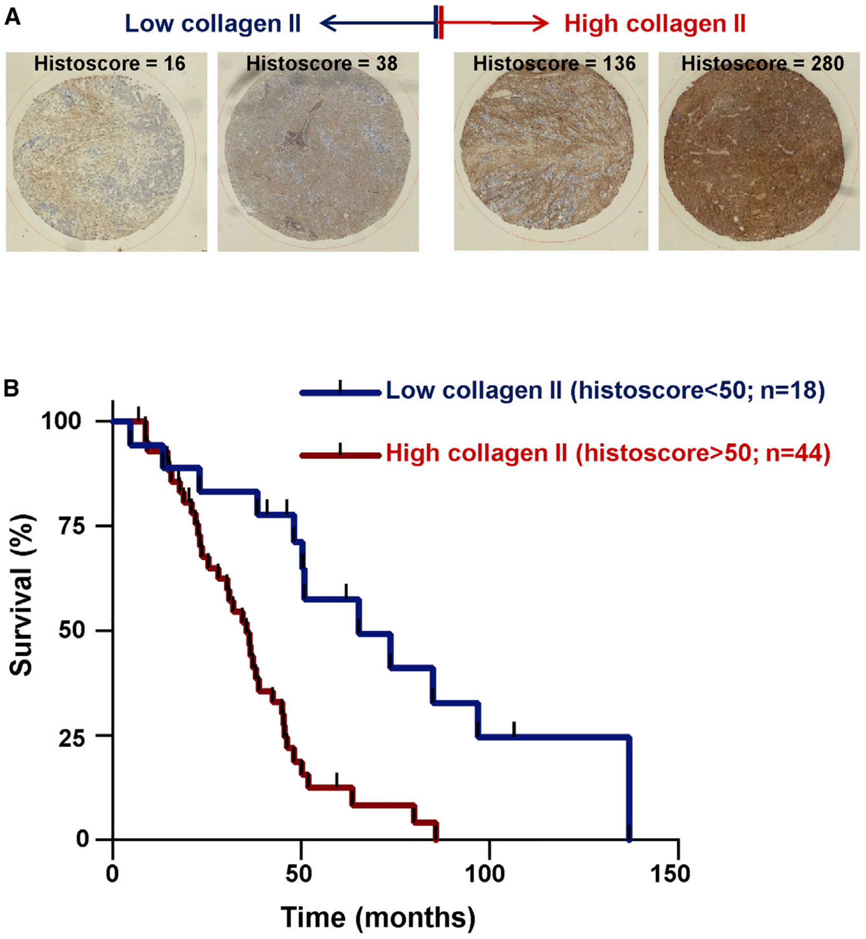

Figure 7. Type II Collagen Expression Predicts Poor Prognosis in Ovarian Cancer

A tissue microarray (TMA) containing 62 cases of ovarian cancer was stained for type II collagen. Examples of tumor cores displaying low (left side) and high (right side) histoscores for collagen II are displayed in (A). (B) shows a Kaplan-Meier analysis of patient survival when the cohort is divided into tumors with low (histoscore < 50) and high (histoscore > 50) collagen II expression. Patients with tumors displaying a collagen II histoscore greater than 50 have a 2.992-fold increased hazard ratio for death; $p = 0.0002$ (log rank test); $n = 62$. See Figure S7 for Kaplan-Meier analysis of the relationship between collagen II levels in the high-grade serous subset of these tumors.

Targeting the translational initiation machinery with drugs has been shown to have sufficiently selective effects on protein synthesis in tumor cells to influence cancer progression. Indeed, eIF4A, which is the molecular target for the promising anticancer agent silvestrol, favors translation of key oncoproteins such as Myc, Notch, and BCL2 [22]. Our findings indicate that control of $tRNA_i^{Met}$ levels in stromal fibroblasts is key to the generation of a collagen-II-rich pro-tumorigenic microenvironment, and this will provide further impetus to the search for drugs to manipulate translation initiation and ECM synthesis in cancer.

Our data also have implications for understanding the mechanisms of tumor angiogenesis. Although stromal fibroblasts are known to contribute to angiogenesis in tumors, the mechanisms through which fibroblasts promote tumor angiogenesis are incompletely understood [23]. The data presented here suggest that the secretion of a pro-migratory ECM by stromal fibroblasts is an important mechanism via which stromal fibroblasts promote angiogenesis in cancer.

EXPERIMENTAL PROCEDURES

Cell Culture

Primary MEFs were isolated from embryonic day 13 (E13)–E15 WT or 2+$tRNA_i^{Met}$ embryos. After sterile dissection from the yolk sac and removal of the fetal liver and head, the embryo was disaggregated by forceful pipetting and cells pelleted by centrifugation and resuspended in DMEM supplemented with 10% FBS, 200 μM L-glutamine, 100 U/ml penicillin streptomycin, 0.25 μg/ml Fungizone, and 50 μM β-mercaptoethanol. Cells were cultured on 0.1% gelatin-coated plates and used as described in the text. For routine culture, human umbilical vein endothelial cells (HUVECs) from pooled donors (TCS Cell Works) were seeded onto 0.1% gelatin-coated plates and cultured in M199 medium supplemented with 20% FBS, 0.1 mg/ml bovine brain endothelial mitogen, 1 ng/ml heparin, and 100 U/ml penicillin streptomycin. HUVECs were used for experiments at passages 4–8. Both B16F0 and LLC cells were cultured in DMEM supplemented with 10% FBS.

Animal Experimentation

All animal work was carried out with ethical approval (University of Glasgow and Institute of Cancer Research) in dedicated barrier animal facilities proactive in environmental enrichment and in accordance with the revised Animals (Scientific Procedures) Act 1986 and the EU Directive 2010/63/EU.

2+$tRNA_i^{Met}$ Mice

Two copies of the murine $tRNA_i^{Met}$ gene (tRNA78), including ~140 bp of flanking sequence, were targeted into the HPRT locus of HM1 embryonic stem cells and used to generate chimeric mice. 2+$tRNA_i^{Met}$ transgenic offspring were derived by crosses to C57Bl/6 mice and maintained on a C57Bl6/J background.

Syngeneic Allografts

Cells were injected subcutaneously into the right flank of either WT or 2+$tRNA_i^{Met}$ transgenic mice. Tumors were harvested at 21 or 22 days post-implantation (for LLC and B16) or at endpoint (for melanocytes), and final tumor volume was calculated using the formula $(L \times W \times W)/2$, where L is the longest dimension and W is the shortest dimension. For blood vessel identification, tumors were fixed and stained for endomucin. Vessels were counted across the entire tumor section and are reported as vessels/mm^2. Only tumors of a similar size were counted, with extremely large and extremely small tumors excluded to allow for reasonable biological comparison. Selection of tumors to analyze was made before blood vessel counts. Forty milligrams per kilogram DHB was administered daily by intraperitoneal injection.

Expression Vectors

Fragments containing $tRNA_i^{Met}$ were initially cloned into the multiple cloning site of the pLPCX vector using *Eco*RI. The pLPCX-$tRNA_i^{Met}$ construct and pLHCX final destination vector were digested using *Bgl*II and *Cla*I and ligated to produce the pLHCX-$tRNA_i^{Met}$-containing vector. The vector was digested with *Hpa*I and *Sna*BI and then re-ligated. For CRISPR gene editing,

single-stranded oligonucleotides were designed to exon 1 of collagen II, annealed to make double-stranded oligos, and ligated into the linearized GeneArt CRISPR Nuclease Vector as per manufacturer's instructions (Life Technologies).

qRT-PCR

RNA was prepared using Trizol (Life Technologies) and cDNA prepared using Quantitech Reverse Transcription Kit (QIAGEN), both as per manufacturer's instructions, using 5, 10, and 20 ng RNA templates to ensure the reaction was quantitative. qPCR was conducted using SyBr green (Quanta) and the Bio-Rad CFX platform, with a 60°C annealing temperature and the primer pairs that are tabulated in the Supplemental Experimental Procedures.

siRNA Transfection

iMEFs were transfected with 10 nM collagen II siRNA (L-043139-01-0005 mouse Col2a1 SMARTpool; Dharmacon), or non-targeting siRNA, using the Amaxa Nucleofector system, solution R, program T-20, as per manufacturer's instructions (Lonza). Cells were used for experiments at 48 hr post-transfection.

ECM Generation and Cell Migration

The preparation of cell-derived matrix was performed as previously described [14], as was time-lapse analysis of cell migration [13].

AFM

To measure the thickness of ECMs following wound scratch, AFM force spectroscopy and contact imaging were performed using a JPK NanoWizard II Bio AFM in combination with a Bruker MLCT cantilever. A Nanoworld Arrow TL-1 cantilever with bead attached was used for force spectroscopy.

SILAC Mass Spectrometry

iMEF-vector and iMEF-$tRNA_i^{Met}$ cells were labeled with light (Arg0/Lys0; Sigma) and heavy amino acids (Arg10/Lys8; Cambridge Isotopes Laboratories). In the case of the forward experiment, iMEF-vector cells were labeled with light amino acids and iMEF-$tRNA_i^{Met}$ cells with heavy amino acids, whereas in the reverse experiment, iMEF-vector cells were labeled with heavy amino acids and iMEF-$tRNA_i^{Met}$ cells with light amino acids. Briefly, cells were passaged in SILAC DMEM containing light or heavy amino acids supplemented with 10% FBS for three passages and then transferred to media containing light or heavy amino acids supplemented with 10% dialyzed FBS. Cells were cultured for 48 hr at 37°C/5% CO_2, washed twice with PBS, and transferred into serum-free media for 6 hr at 37°C/5% CO_2. The media was then acidified to pH 5 with 10% TFA and 10 μl Strataclean beads added per 1 ml media. Beads were collected by brief centrifugation and proteins separated by electrophoresis. Proteins were digested with 0.05 μg/ml trypsin in 50 mM ammonium bicarbonate overnight. Extracted digests were stage tipped and analyzed on a linear trap quadrupole (LTQ)-Orbitrap Elite. Data were searched and quantified against Uniprot MOUSE using MaxQuant [24].

Tissue Microarray and Immunohistochemistry

The SaPPrOC trial (Clinicaltrials.gov reference NCT01196741) was a randomized phase II study of weekly paclitaxel ± oral saracatinib in women with relapsed, platinum-resistant ovarian cancer. The study was approved by the UK National Research Ethics Service (reference 10/H1211/26), and the primary outcome data are reported elsewhere [25]. A tissue microarray was created with triplicate 5-mm cores from formalin-fixed paraffin-embedded tumor material collected at the time of diagnosis. Five-micrometer sections from formalin-fixed paraffin-embedded tumors were stained for collagen II expression (Dako Autostainer). Stained slides were digitized (Hamamatsu NanoZoomer NDP; Hamamatsu Photonics) and viewed using Slidepath Digital Image Hub V4.0.7 (Leica Microsystems). Areas of tumor were identified and scored using Slidepath Tissue Image Analysis and histoscores generated by multiplying intensity of cellular staining within marked areas (range 0–3) by percentage tumor cells with positive staining (range 0–100), with a maximum histoscore of 300. Data represent mean histoscore per tumor.

Ethics Approval for the Use of Patient-Derived Material

Use of breast cancer patient samples is covered by Research Tissue Bank Ethics approval (reference: East of England REC 15/EE/0413) and follows informed patient consent and approval by Tissue Access Committee. All ovarian cancer samples were collected following informed patient consent under the auspices of NHS Greater Glasgow and Clyde Biorepository following approval by West of Scotland Research Ethics Committee 4 (reference: 10/S0704/60).

SUPPLEMENTAL INFORMATION

Supplemental Information includes seven figures, Supplemental Experimental Procedures, and two tables and can be found with this article online at http://dx.doi.org/10.1016/j.cub.2016.01.045.

AUTHOR CONTRIBUTIONS

C.J.C., T.J.B., A.R.R., S.Z., and J.C.N. designed the experiments. C.J.C., T.J.B., A.R.R., L.M., D.E., C.C., D. Sumpton, C.N., K.C., V.L.B., S.F., P.B.V., E.P., E.K., and D. Strathdee performed the experiments. C.J.C., J.C.N., and A.R.R. wrote the paper. C.J.C., J.C.N., A.R.R., T.J.B., D.E., and I.M. analyzed the data.

ACKNOWLEDGMENTS

This work was generously supported by funding from Cancer Research UK—grant numbers C596/A17196 (core), C596/A12935 (to S.Z.), and C596/A18277 (to J.N.). A.R.R., S.F., E.K., and V.L.B. were supported by funding from Breakthrough Breast Cancer (recently merged with Breast Cancer Campaign, forming Breast Cancer Now). We thank the Breast Cancer Now Tissue Bank Cell Culture Programme for the supply of primary human cells. The authors thank the staff of the Beatson Institute and ICR biological services units and the Beatson Institute and Breakthrough Breast Cancer histopathology core facilities for technical assistance.

Received: July 9, 2015
Revised: December 7, 2015
Accepted: January 19, 2016
Published: March 3, 2016

REFERENCES

1. Dittmar, K.A., Goodenbour, J.M., and Pan, T. (2006). Tissue-specific differences in human transfer RNA expression. PLoS Genet. *2*, e221.
2. Gingold, H., Tehler, D., Christoffersen, N.R., Nielsen, M.M., Asmar, F., Kooistra, S.M., Christophersen, N.S., Christensen, L.L., Borre, M., Sørensen, K.D., et al. (2014). A dual program for translation regulation in cellular proliferation and differentiation. Cell *158*, 1281–1292.
3. Pavon-Eternod, M., Gomes, S., Geslain, R., Dai, Q., Rosner, M.R., and Pan, T. (2009). tRNA over-expression in breast cancer and functional consequences. Nucleic Acids Res. *37*, 7268–7280.
4. Zhou, Y., Goodenbour, J.M., Godley, L.A., Wickrema, A., and Pan, T. (2009). High levels of tRNA abundance and alteration of tRNA charging by bortezomib in multiple myeloma. Biochem. Biophys. Res. Commun. *385*, 160–164.
5. Mahlab, S., Tuller, T., and Linial, M. (2012). Conservation of the relative tRNA composition in healthy and cancerous tissues. RNA *18*, 640–652.
6. Pavon-Eternod, M., Gomes, S., Rosner, M.R., and Pan, T. (2013). Overexpression of initiator methionine tRNA leads to global reprogramming of tRNA expression and increased proliferation in human epithelial cells. RNA *19*, 461–466.
7. Rideout, E.J., Marshall, L., and Grewal, S.S. (2012). *Drosophila* RNA polymerase III repressor Maf1 controls body size and developmental timing by modulating tRNAiMet synthesis and systemic insulin signaling. Proc. Natl. Acad. Sci. USA *109*, 1139–1144.
8. Loreni, F., Mancino, M., and Biffo, S. (2014). Translation factors and ribosomal proteins control tumor onset and progression: how? Oncogene *33*, 2145–2156.

9. Gao, B., and Roux, P.P. (2015). Translational control by oncogenic signaling pathways. Biochim. Biophys. Acta *1849*, 753–765.

10. Duluc, C., Moatassim-Billah, S., Chalabi-Dchar, M., Perraud, A., Samain, R., Breibach, F., Gayral, M., Cordelier, P., Delisle, M.B., Bousquet-Dubouch, M.P., et al. (2015). Pharmacological targeting of the protein synthesis mTOR/4E-BP1 pathway in cancer-associated fibroblasts abrogates pancreatic tumour chemoresistance. EMBO Mol. Med. *7*, 735–753.

11. Kojima, Y., Acar, A., Eaton, E.N., Mellody, K.T., Scheel, C., Ben-Porath, I., Onder, T.T., Wang, Z.C., Richardson, A.L., Weinberg, R.A., and Orimo, A. (2010). Autocrine TGF-beta and stromal cell-derived factor-1 (SDF-1) signaling drives the evolution of tumor-promoting mammary stromal myofibroblasts. Proc. Natl. Acad. Sci. USA *107*, 20009–20014.

12. Lindsay, C.R., Lawn, S., Campbell, A.D., Faller, W.J., Rambow, F., Mort, R.L., Timpson, P., Li, A., Cammareri, P., Ridgway, R.A., et al. (2011). P-Rex1 is required for efficient melanoblast migration and melanoma metastasis. Nat. Commun. *2*, 555.

13. Caswell, P.T., Chan, M., Lindsay, A.J., McCaffrey, M.W., Boettiger, D., and Norman, J.C. (2008). Rab-coupling protein coordinates recycling of alpha5beta1 integrin and EGFR1 to promote cell migration in 3D microenvironments. J. Cell Biol. *183*, 143–155.

14. Cukierman, E., Pankov, R., Stevens, D.R., and Yamada, K.M. (2001). Taking cell-matrix adhesions to the third dimension. Science *294*, 1708–1712.

15. van den Biggelaar, M., Hernández-Fernaud, J.R., van den Eshof, B.L., Neilson, L.J., Meijer, A.B., Mertens, K., and Zanivan, S. (2014). Quantitative phosphoproteomics unveils temporal dynamics of thrombin signaling in human endothelial cells. Blood *123*, e22–e36.

16. Gilkes, D.M., Chaturvedi, P., Bajpai, S., Wong, C.C., Wei, H., Pitcairn, S., Hubbi, M.E., Wirtz, D., and Semenza, G.L. (2013). Collagen prolyl hydroxylases are essential for breast cancer metastasis. Cancer Res. *73*, 3285–3296.

17. Hall, M., Gourley, C., McNeish, I., Ledermann, J., Gore, M., Jayson, G., Perren, T., Rustin, G., and Kaye, S. (2013). Targeted anti-vascular therapies for ovarian cancer: current evidence. Br. J. Cancer *108*, 250–258.

18. Qian, W., Yang, J.R., Pearson, N.M., Maclean, C., and Zhang, J. (2012). Balanced codon usage optimizes eukaryotic translational efficiency. PLoS Genet. *8*, e1002603.

19. Manojlovic, Z., and Stefanovic, B. (2012). A novel role of RNA helicase A in regulation of translation of type I collagen mRNAs. RNA *18*, 321–334.

20. Cheng, J.C., and Leung, P.C. (2011). Type I collagen down-regulates E-cadherin expression by increasing PI3KCA in cancer cells. Cancer Lett. *304*, 107–116.

21. Zou, X., Feng, B., Dong, T., Yan, G., Tan, B., Shen, H., Huang, A., Zhang, X., Zhang, M., Yang, P., et al. (2013). Up-regulation of type I collagen during tumorigenesis of colorectal cancer revealed by quantitative proteomic analysis. J. Proteomics *94*, 473–485.

22. Wolfe, A.L., Singh, K., Zhong, Y., Drewe, P., Rajasekhar, V.K., Sanghvi, V.R., Mavrakis, K.J., Jiang, M., Roderick, J.E., Van der Meulen, J., et al. (2014). RNA G-quadruplexes cause eIF4A-dependent oncogene translation in cancer. Nature *513*, 65–70.

23. Kalluri, R., and Zeisberg, M. (2006). Fibroblasts in cancer. Nat. Rev. Cancer *6*, 392–401.

24. Cox, J., and Mann, M. (2008). MaxQuant enables high peptide identification rates, individualized p.p.b.-range mass accuracies and proteome-wide protein quantification. Nat. Biotechnol. *26*, 1367–1372.

25. McNeish, I.A., Ledermann, J.A., Webber, L., James, L., Kaye, S.B., Hall, M., Hall, G., Clamp, A., Earl, H., Banerjee, S., et al. (2014). A randomised, placebo-controlled trial of weekly paclitaxel and saracatinib (AZD0530) in platinum-resistant ovarian, fallopian tube or primary peritoneal cancer. Ann. Oncol. *25*, 1988–1995.

Current Biology, Volume *26*

Supplemental Information

The Initiator Methionine tRNA Drives Secretion of Type II Collagen from Stromal Fibroblasts to Promote Tumor Growth and Angiogenesis

Cassie J. Clarke, Tracy J. Berg, Joanna Birch, Darren Ennis, Louise Mitchell, Catherine Cloix, Andrew Campbell, David Sumpton, Colin Nixon, Kirsteen Campbell, Victoria L. Bridgeman, Peter B. Vermeulen, Shane Foo, Eleftherios Kostaras, J. Louise Jones, Linda Haywood, Ellie Pulleine, Huabing Yin, Douglas Strathdee, Owen Sansom, Karen Blyth, Iain McNeish, Sara Zanivan, Andrew R. Reynolds, and Jim C. Norman

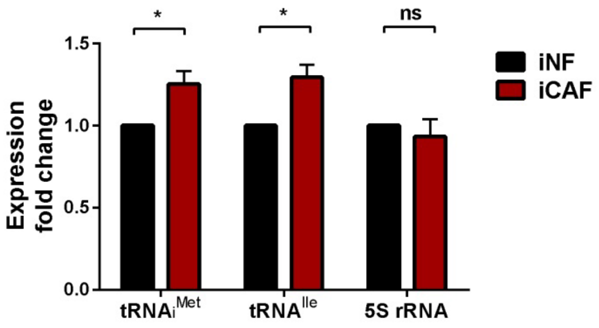

Figure S1. Changes in expression of specific tRNAs in cancer associated fibroblasts compared to normal fibroblasts.

qPCR was used to quantify expression of $tRNA_i^{Met}$, $tRNA^{Ile}$, and 5S rRNA in immortalised normal fibroblasts (iNF) and immortalised cancer associated fibroblasts (iCAF). All samples were normalised to ARPP P0 and are presented relative to iNF, n=minimum of 3, ± SEM, unpaired t-test, * $p < 0.05$.

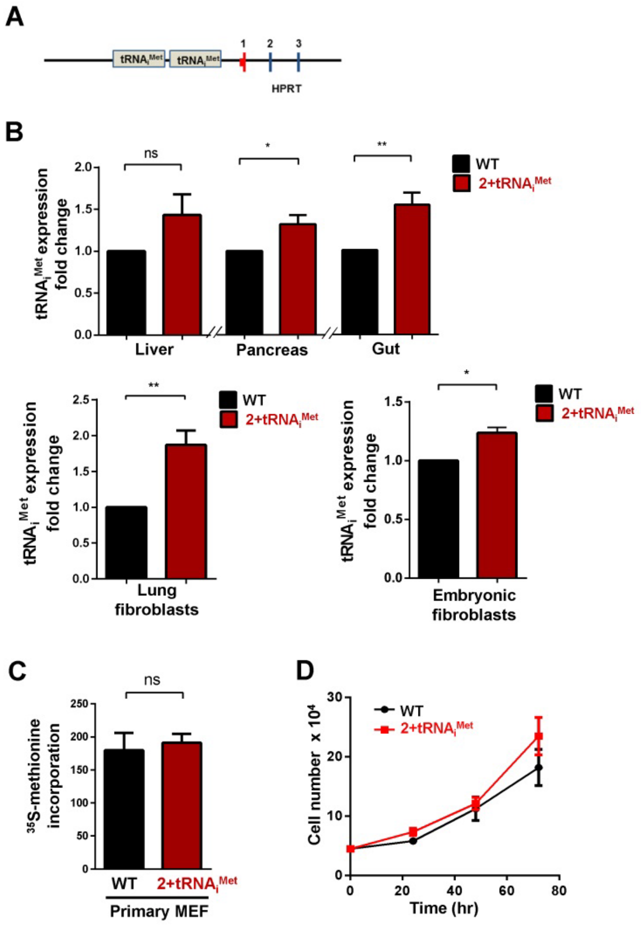

Figure S2. Characterisation of cells from $2+tRNA_i^{Met}$ mouse (related to Figure 2)

(A) The $2+tRNA_i^{Met}$ mouse was engineered to express two additional copies of the $tRNA_i^{Met}$ transgene in the HPRT locus on the X-chromosome.

(B) qPCR of $tRNA_i^{Met}$ expression in primary fibroblasts isolated from the lungs and embryos of the $2+tRNA_i^{Met}$ mouse. All values are normalised to expression of ARPP P0, n=4 lung and n=2 embryonic fibroblasts, ± SEM, unpaired t-test, * $p < 0.05$. ** $p < 0.005$.

(C) ^{35}S-methionine incorporation was used to assess the synthesis of new proteins. Primary cultured MEFs were depleted of methionine and cysteine for 30 minutes at 37°C/5% CO_2, then trypsinised and resuspended in media containing 0.07mCi EasyTag Express Protein Labelling Max ^{35}S and incubated at 37°C/5% CO_2 for 2 hours. TCA precipitation was used to stop and concentrate the reaction, and the ratio of TCA precipitated radiolabel to total radiolabel calculated, values are mean ± SEM, n=5.

(D) Primary cultured MEFs were isolated from wild-type and $2+tRNA_i^{Met}$ embryos, plated onto plastic dishes and their proliferation rate was determined over an 72 hr period. Values are mean ± SEM, n=2.

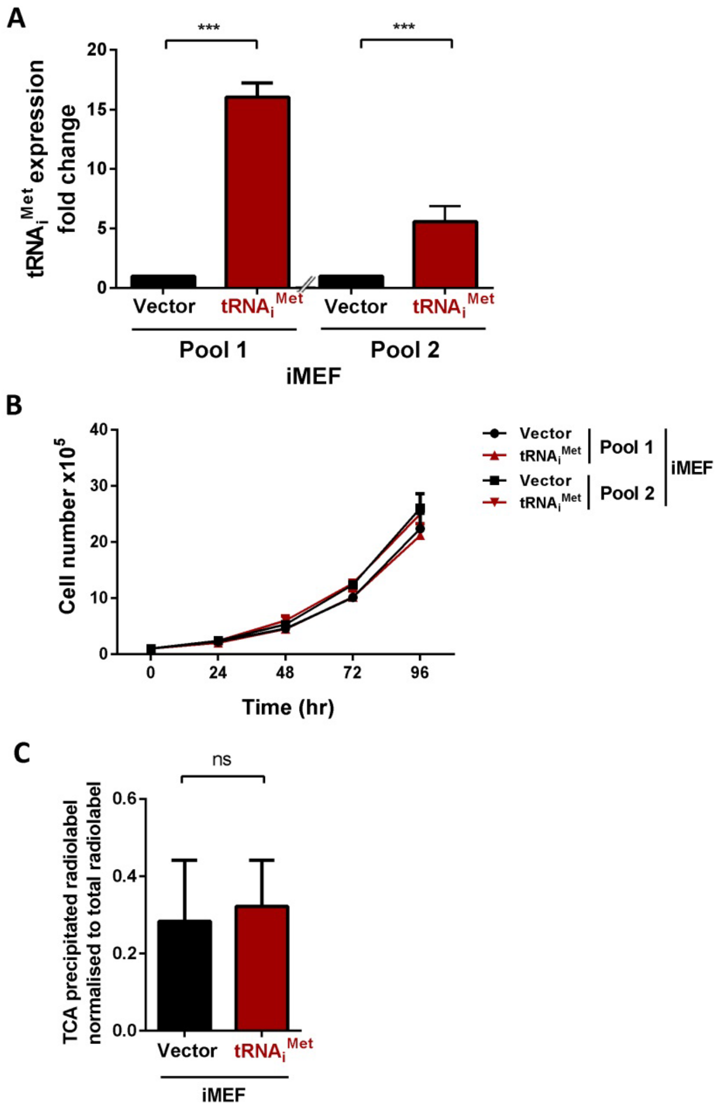

Figure S3. Characterisation of $tRNA_i^{Met}$ overexpressing fibroblasts (related to Figures 3 & 4)

(A) qPCR of $tRNA_i^{Met}$ expression in pools of immortalised mouse embryonic fibroblasts. Values are normalised to expression of ARPP P0, n=3, ± SEM, unpaired t-test, *** $p < 0.0005$.

(B) Two pools of immortalised iMEFs which were overexpressing $tRNA_i^{Met}$ or vector control were plated onto plastic dishes and their proliferation rate was determined over an 96 hr period. Values are mean ± SEM, n=3.

(C) Immortalised MEFs which were overexpressing $tRNA_i^{Met}$ or vector control were labelled with ^{35}S-methionine and the incorporation of the tracer into newly synthesised protein was determined as for Figure S2C. Values are mean ± SEM, n=5.

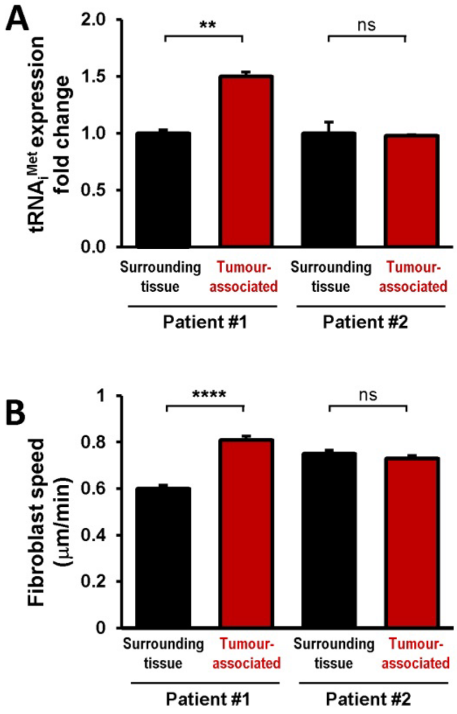

Figure S4. The relationship between tRNA$_i^{Met}$ levels and the migratory properties of ECM deposited by primary cultured CAFs.

Carcinoma-associated fibroblasts (CAFs) and control fibroblasts from surrounding normal tissue were isolated from resected material from two breast cancer patients (patient #1 and patient #2). qPCR was used to quantify tRNA$_i^{Met}$ levels (A). ECM was generated from these two pairs of carcinoma associated and normal fibroblasts and iMEFs were plated onto these. The speed of fibroblast migration on these ECMs was determined (B). Values are mean ± SEM, unpaired t-test, *** p <0.0001, ** p <0.001.

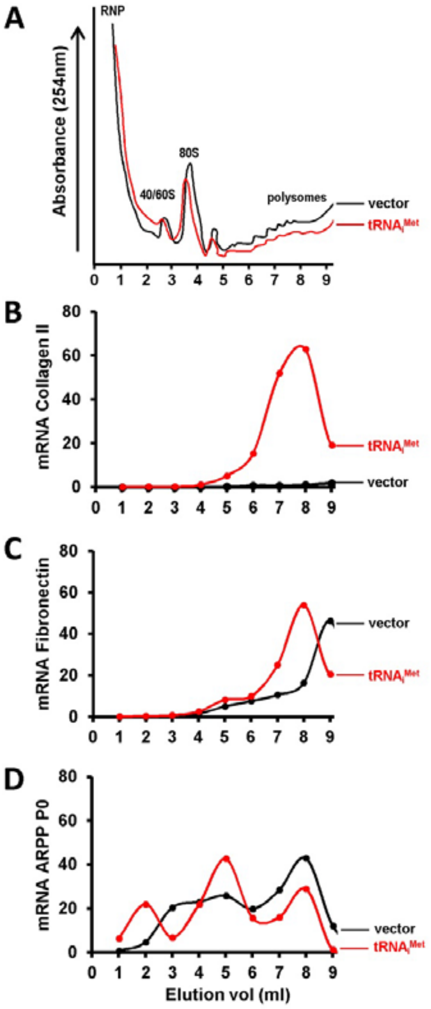

Figure S5. Polysome analysis of tRNA$_i^{Met}$ overexpressing fibroblasts (related to Figure 4)

iMEF-vector and iMEF-tRNA$_i^{Met}$ cells were treated with cycloheximide for 3 min and then lysed on ice. Lysates were centrifuged and loaded onto sucrose density gradients and centrifuged at 38,000rpm for 2 hr. Fractions were eluted (A) from the gradient and the presence of mRNAs for collagen II (B), fibronectin (C) and ARPP P0 (D) were determined using qPCR.

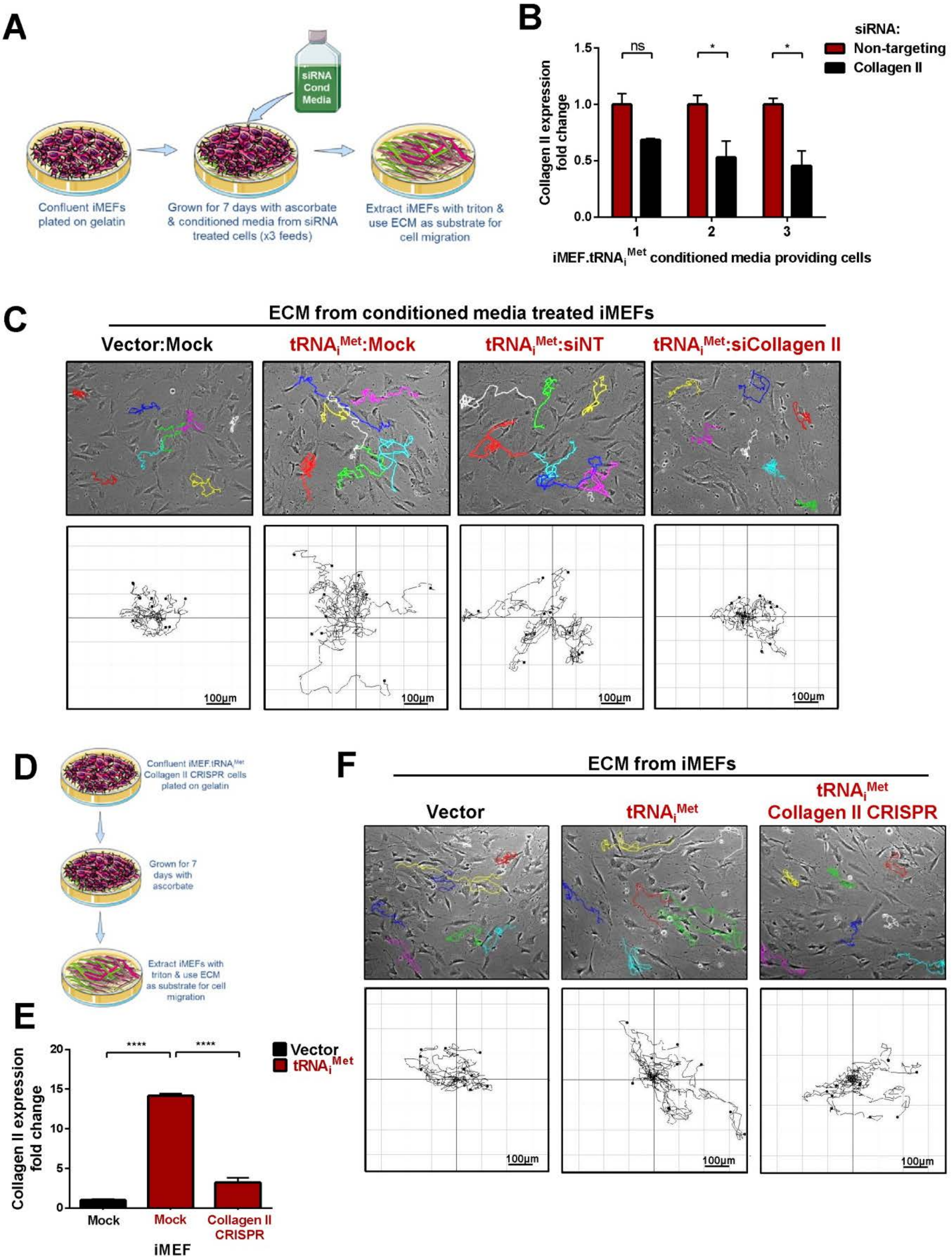

Figure S6. Type II Collagen secretion is required for $tRNA_i^{Met}$ to drive production of a pro-tumourigenic ECM (related to Figure 6)

(A) Schematic representation of the method used to generate conditioned media-derived ECM. iMEF cells were plated to confluence on gelatin-coated plates, and then grown for a further 7 days in ascorbate containing media that was diluted 1:1 with filter-sterilised conditioned media from iMEF.$tRNA_i^{Met}$ cells treated with either non-targeting or type II collagen-specific siRNA. The ascorbate media/conditioned media mix was refreshed and replaced every 2 days over a 7 day period. Cells were then removed using a triton containing buffer, leaving the ECM of interest coating the culture dish.

(B) qPCR was used to quantify type II collagen knockdown in the cells providing conditioned media to generate ECM. Values are normalised to expression of ARPP P0 and presented relative to expression in the iMEF.tRNA$_i^{Met}$ non-targeting siRNA-treated cells, values are mean ± SD, n=3, *p<0.05.

(C) Representative cell tracks and spider plots corresponding to the data displayed in Figure 5A.

(D) Schematic representation of method used to generate ECM from iMEF pools. iMEF cells were plated to confluence on gelatin-coated plates, and then grown for a further 7 days in ascorbate containing media. The ascorbate containing media was refreshed and replaced every 2 days over a 7 day period. Cells were then removed using a triton containing buffer, leaving the ECM of interest coating the culture dish.

(E) qPCR was used to quantify type II collagen levels in iMEF-vector and iMEF-tRNA$_i^{Met}$ cells transfected with an empty CRISPR vector, and in iMEF-tRNA$_i^{Met}$ cells transfected with a CRISPR vector specific for type II collagen. Values are normalised to expression of ARPP P0 and presented relative to expression in the iMEF.vector cells.

(F) Representative cell tracks and spider plots corresponding to the data displayed in Figure 5C.

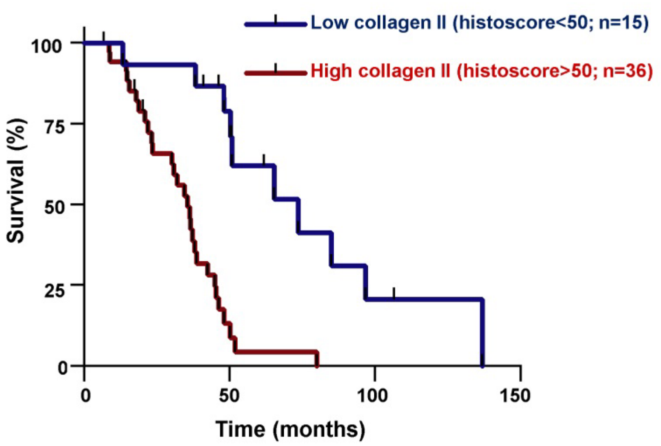

Figure S7. Type II collagen expression predicts poor prognosis in high grade serous ovarian cancer (related to Figure 7)

A tissue microarray (TMA) containing 51 cases of high grade serous ovarian cancer was stained for type II collagen and analysed as for Figure 7. The Kaplan-Meier analysis patient survival when the cohort is divided into tumours with low (histoscore < 50) and high (histoscore >50) collagen II expression. Patients with tumours displaying a collagen II histoscore greater than 50 have a 3.818 fold increased hazard ratio for death, p = 0.0001 (logrank test), n = 51.

SUPPLEMENTAL EXPERIMENTAL PROCEDURES

Polysome gradient analysis and qPCR. Cycloheximide (Sigma) was added for 3 minutes to the cells at 37°C. Two washes in ice-cold PBS containing cycloheximide were used to detach the cells from plates and then the cells were pelleted. Cell pellet were resuspended in ice cold lysis buffer (0.3M NaCl, 15mM MgCl2, 15mM Tris-HCl pH 7.5, 0.1mg/ml CHX, 1% Triton-X100). Lysates were centrifuged for 1 minute at 13 000rpm and the supernatant loaded onto a 10-50% sucrose gradient and subjected to ultracentrifugation at 38 000 rpm for 2 hours. Gradients were eluted using a Teledyne Isco density gradient fractionation system composed of UA-6 detector and optical unit, and a Foxy R1 fraction collector. RNAs were extracted from fractions using Trizol® (ThermoFisher Scientific) according to manufacturer's instructions. First strand cDNA synthesis was performed with equivalent volumes of extracted total RNA from each fraction was carried out using Quantitect® reverse transcription kit (Qiagen) according to manufacturer's instructions. For qPCR, cDNA was diluted 2-fold and 3 µl of this was used for analysis using PerfeCTa® SYBR® Green Fast Mix® (Quanta Biosciences). Quantitative PCR, carried out in a Bio-Rad CFX96, was performed thus; 95°C for 3 min followed by 40 cycles at 95°C for 20 s, 60°C for 20s and 72°C for 20s followed by a dissociation curve.

Isolation of primary cultured fibroblasts from breast cancer patients. Primary human breast fibroblasts were isolated from normal (reduction mammoplasty), tumour and surround (>5cm from tumour edge) tissue samples, following patient consent. Tissue was digested for 12 to 16 hours at 37˚C in RPMI-1640 medium plus 25mM Hepes, supplemented with 5% foetal bovine serum (FBS), penicillin (100U/ml), streptomycin (0.1mg/ml) and amphotericin-B (5µg/ml) containing 1mg/ml collagenase 1A and hyaluronidase on a rotary shaker. The digested tissue was centrifuged at 380g for 20 minutes and washed in medium three times to remove enzymes. The normal and surround tissue isolates were then sedimented three times at 1g for 30 mins to collect the denser organoids. The supernatants containing the fibroblasts were centrifuged (380g x 3minutes) and the cell pellets re-suspended and cultured in DMEM:F12 supplemented with 10% FBS and penicillin/streptomycin and amphotericin-B as above. Following centrifugation and washing steps, digested tumour tissue was filtered through a 40µm filter and the filtrate centrifuged and cultured as for the normal and surround cell isolates. Isolated cells were cultured for two to three passages prior to conducting experiments to guarantee the purity of the fibroblast populations.

Primer pairs used for qRT-PCR

Primer name	Primer Sequence 5' – 3'	Primer name	Primer Sequence 5' – 3'
$tRNA_i^{Met}$ Forward	AGAGTGGCGCAGCGGAAG	$tRNA_i^{Met}$ Reverse	AGCAGAGGATGGTTTCGATCC
$tRNA^{Ile}$ Forward	GGCGGCCGGTTAGCTCAG	$tRNA^{Ile}$ Reverse	CCCGTACGGGGATCGAAC
5S rRNA Forward	CAGCACCCGGTATTCCCAGG	5S rRNA Reverse	GGCATACCACCCTGAACGC
Collagen II Forward	GGGTCACAGAGGTTACCCAG	Collagen II Reverse	ACCAGGGGAACCACTCTCAC
ARPP P0 Forward	GCACTGGAAGTCCAACTACTTC	ARPP P0 Reverse	TGAGGTCCTCCTTGGTGAACAC
